# Application of deep learning for real-time detection, localization, and counting of the malignant invasive weed Solanum rostratum Dunal

**DOI:** 10.3389/fpls.2024.1486929

**Published:** 2025-01-29

**Authors:** Shifeng Du, Yashuai Yang, Hongbo Yuan, Man Cheng

**Affiliations:** College of Mechanical and Electrical Engineering, Hebei Agricultural University, Baoding, China

**Keywords:** invasive plants, Solanum rostratum Dunal, deep learning, real-time detection, localization, counting

## Abstract

*Solanum rostratum* Dunal (SrD) is a globally harmful invasive weed that has spread widely across many countries, posing a serious threat to agriculture and ecosystem security. A deep learning network model, TrackSolanum, was designed for real-time detection, location, and counting of SrD in the field. The TrackSolanmu network model comprises four modules: detection, tracking, localization, and counting. The detection module uses YOLO_EAND for SrD identification, the tracking module applies DeepSort for multi-target tracking of SrD in consecutive video frames, the localization module determines the position of the SrD through center-of-mass localization, and the counting module counts the plants using a target ID over-the-line invalidation method. The field test results show that for UAV video at a height of 2m, TrackSolanum achieved precision and recall of 0.950 and 0.970, with MOTA and IDF1 scores of 0.826 and 0.960, a counting error rate of 2.438%, and FPS of 17. For UAV video at a height of 3m, the model reached precision and recall of 0.846 and 0.934, MOTA and IDF1 scores of 0.708 and 0.888, a counting error rate of 4.634%, and FPS of 79. Thus, the TrackSolanum supports real-time SrD detection, offering crucial technical support for hazard assessment and precise management of SrD.

## 1 Introduction

Invasive alien plants threaten biodiversity, ecosystem stability, and human health, and require significant local community expenditures for remediation and eradication, causing substantial economic losses (Larson et al., 2020; Yang et al., 2022). *Solanum rostratum* Dunal (SrD) is a globally harmful invasive weed native to North America that has spread to countries including Canada, China, Russia, and Australia (Zhao et al., 2019; Abu-Nassar and Matzrafi, 2021). The leaves, berries, and roots of SrD contain cholinesterase inhibitory compounds, which can cause poisoning and death in livestock when accidentally ingested (Wei et al., 2010). Moreover, SrD is highly drought tolerant, and its berries dry out and split, dispersing thousands of seeds upon maturity. Control and eradication of SrD are challenging, as newly harvested seeds enter dormancy and may germinate under certain conditions (Abu-Nassar et al., 2022), Therefore, real-time detection, localization, and enumeration of this invasive weed are critical for assessing its impact and informing control measures.

Weeds compete with field crops for sunlight, water, nutrients, and space, impeding crop growth and reducing yields. Real-time weed detection is essential to assess invasion levels and provide critical data for effective weed control (Xu et al., 2023). Weed detection is fundamental to implementing control measures, and deep learning techniques are increasingly replacing traditional machine learning methods for real-time weed detection, supported by new models and enhanced computational power (Liu and Bruch, 2020). Effective deep learning-based detection requires robust datasets, and Unmanned Ground Vehicles (UGVs) and Unmanned Aerial Vehicles (UAVs) are two popular platforms for field data collection (Gao et al., 2024). UGVs offer flexibility and adaptability across various terrains as agricultural data collection platforms. Kazmi et al. (2015) collected image data of sugar beet and thistle using UGV and color cameras, detecting thistle with a 97% accuracy using vegetation indices. Wendel et al. (2018) developed a weed detection model for lettuce plots via machine learning, enabling targeted weed removal. Kounalakis et al. (2019) collected weed images using a robotic platform and created a model combining deep learning-based feature extraction with a linear classifier, achieving effective results in field trials. Ju et al. (2024) developed an autonomous rice field weeding robot equipped with the MW-YOLOv5s model to distinguish seedlings from weeds in rice fields.

Compared to ground-based unmanned vehicles, UAV platforms enhance data collection efficiency and provide high-quality image data, forming a solid foundation for training deep learning models. Torres-Sánchez et al. (2021) used artificial neural networks with UGV imagery to detect broad-leaf and grassy weeds in wide-row herbaceous crops, achieving detection accuracies of approximately 75% and 65%, respectively. Su et al. (2022) detected blackgrass weeds in wheat field by integrating UAV-acquired multispectral images and machine learning, with average precision, recall, and accuracy of 93.8%, 93.8%, and 93.0%, respectively. Ong et al. (2023) combined a CNN-based classifier with UAV images to detect weeds in cabbage fields, achieving a recognition accuracy of 92.41%.

Counting plants in the field provides managers with valuable data to inform better management strategies. Deep learning techniques have also been applied to plant counting. Barreto et al. (2021) achieved fully automated plant counting in sugar beet, corn, and strawberry fields using UAV imagery and a fully convolutional network (FCN), reporting counting errors of less than 4.6% for sugar beet and under 4% for corn and strawberries. Chen et al. (2023) proposed the RFF-PC cone counting algorithm based on improved feature fusion, achieving an average counting accuracy of 89.80% on the UFPC 2019 dataset. Li et al. (2024) introduced SoybeanNet, a new point-based counting network that performs both pod counting and high-precision localization, achieving a counting accuracy of 84.51%, which demonstrates its effectiveness in real-world scenarios.

Our team previously utilized the U-Net convolutional network for UAV image analysis to assess the extent of SrD invasion (Wang et al., 2021a). However, the high flight altitude and low image resolution of the UAV limit detection to instances when SrD has reached a certain grown stage, which may already result in ecological damage. To accurately identify and remove the SrD in its early stages, we developed the YOLOv5_CBAM network for detection (Wang et al., 2022). This model was deployed on vehicles operating autonomously in the field for real-time detection. While the effectiveness of SrD detection has been established, the model has not been implemented for accurate counting and localization. Accurate counting is essential for assessing the invasion status of SrD, and localization provides precise location information for targeted remova. Therefore, we designed the deep learning network model TrackSolunam to achieve real-time detection, localization, and counting for SrD. This model can can process videos acquired by UAVs or UGVs in real-time, accurately detecting, tracking, and locating SrD plants while providing accurate counts. This technical support is crucial for evaluating invasion damage and enabling precise management. Additionally, the model’s real-time processing capability allows for faster response to invasive events, facilitating timely preventive and control measures.

The main contributions of this paper are: (1) constructing a comprehensive dataset of the SrD, includes RGB images captured from various devices-such as cameras, smartphones, and UAVs-at different heights and growth stages; (2) developing a deep learning network model, TrackSolanum, which integrates YOLO v8, EMA attention mechanism, and DeepSort to enable real-time detection, localization, and counting of SrD; (3) conducting a field trial using UAV to validate the proposed method.

## 2 Material and method

### 2.1 Data acquisition

Research revealed that there are currently no public datasets specifically for SrD. Therefore, our team conducted image data collection in July 2020, June 2021, and June 2022. The data collection sites were SrD monitoring points in Hebei Province, China, located at the following coordinates: Monitoring Point (MP) 1# (40.42.27N, 114.45.6E), MP 2# (40.39.58N, 114.46.12E), MP 3# (40.47.6N, 114.42.7E), and MP 4# (40.43.5N, 114.43.13E). At each site, SrD was in its natural growth state. Image capture devices included a NIKON D610 (Nikon, Tokyo, Japan), a smartphone (HUAWEI Nova 3, Shenzhen, China), and a MAVIC AIR (DJI, Shenzhen, China). The NIKON D610 and smartphone images were taken from approximately 1.2 meters above ground, while the MAVIC AIR captured images from altitudes of 2, 3, and 5 meters. A total of 513 valid images were collected: 222 images from the NIKON D610, 150 images from the smartphone, 45 images from the MAVIC AIR at 2meters, 44 images at 3 meters, and 52 images at 5 meters. At MP 4#, video data were captured using UAV from heights of 2 and 3 meters. The video data collected were effective for 143 seconds at a two-meter height and 122 seconds at three-meter height. The choice of shooting heights for different devices was based on our team’s previous research, which showed that images captured at these heights effectively highlight the botanical characteristics of SrD for identification. The images and videos obtained cover multiple growth stages of SrD, including the seedling, growth stage, and flowering and fruiting stage. **Table 1** provides details on the acquisition devices and images resolutions.

**Table 1:** Imaging devices, resolutions and image examples.

Imaging devices	Resolution	Image examples		
NIKON D610	6016×4016	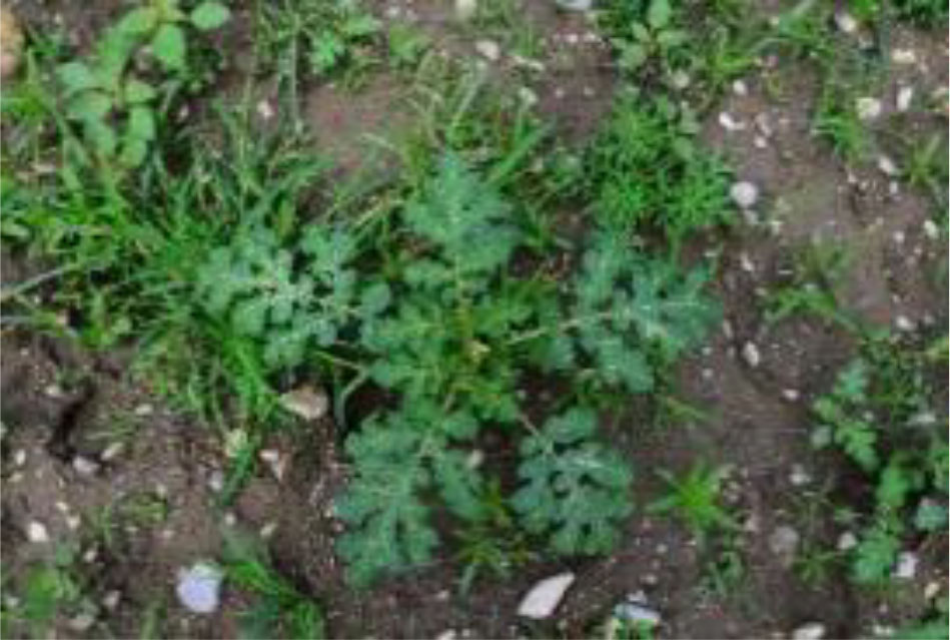	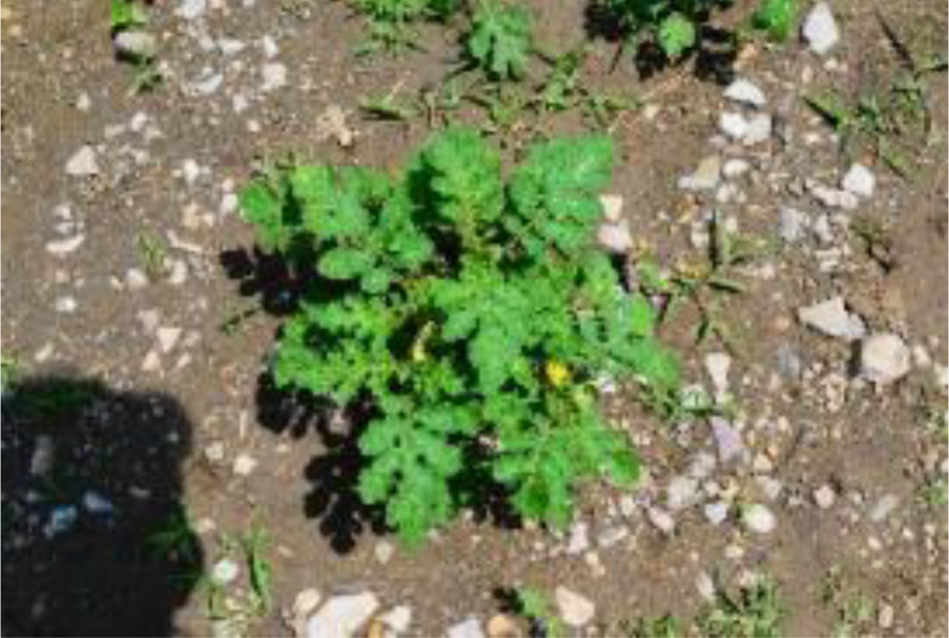	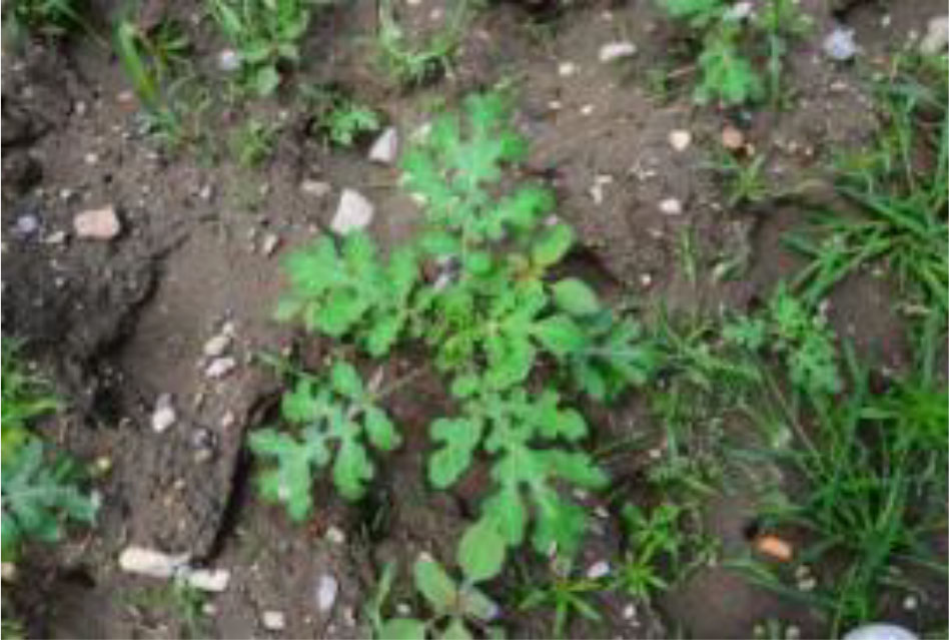
Mobile phone	4608×3456	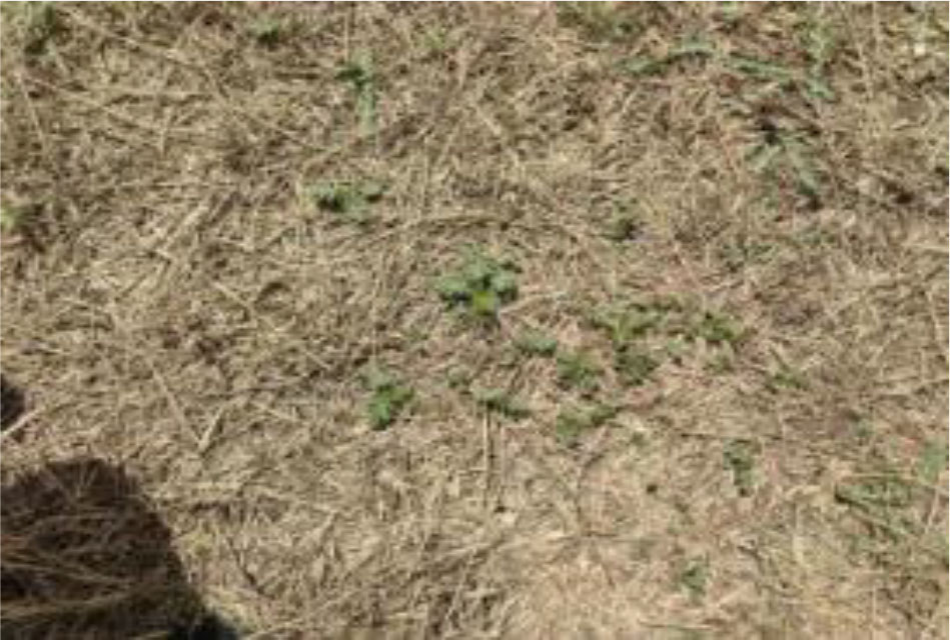	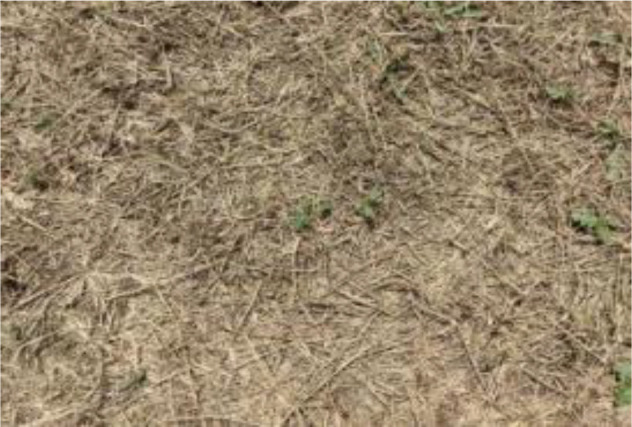	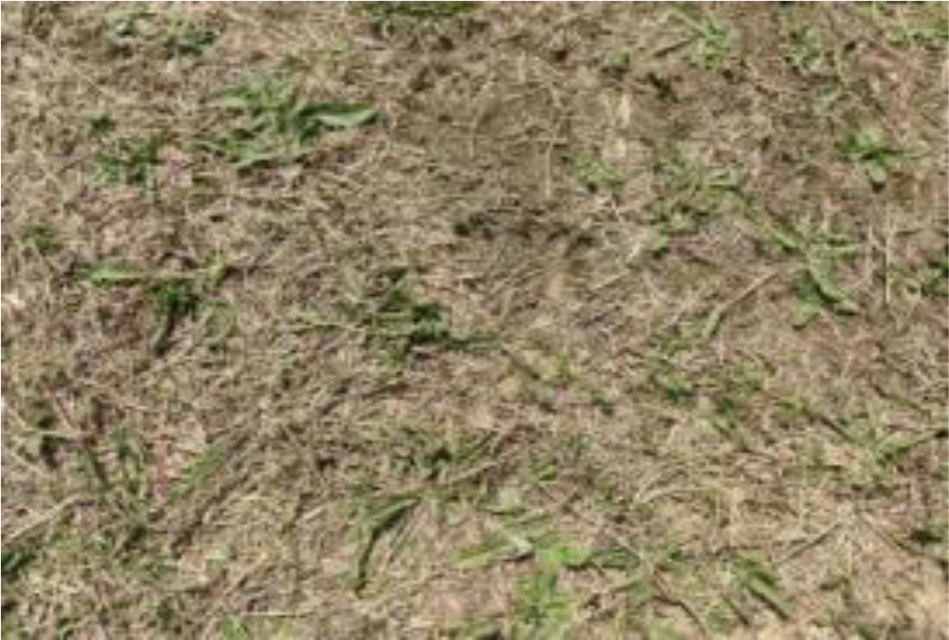
MAVIC AIR	4056×3040	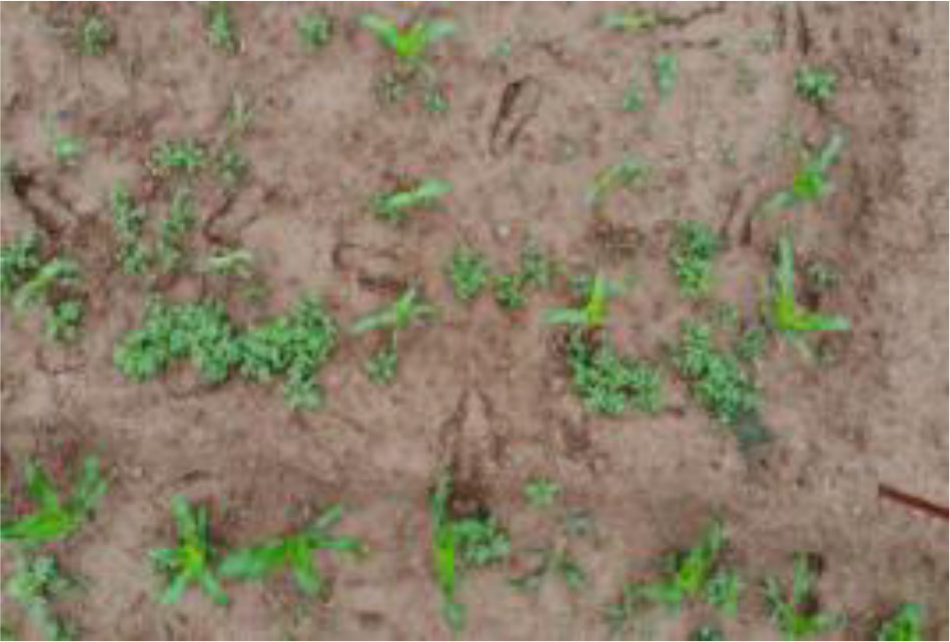	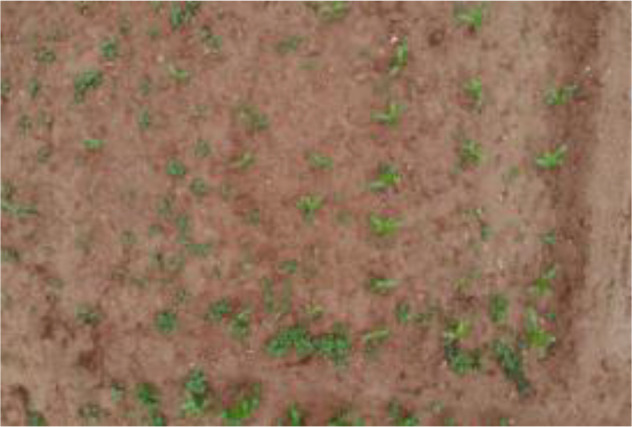	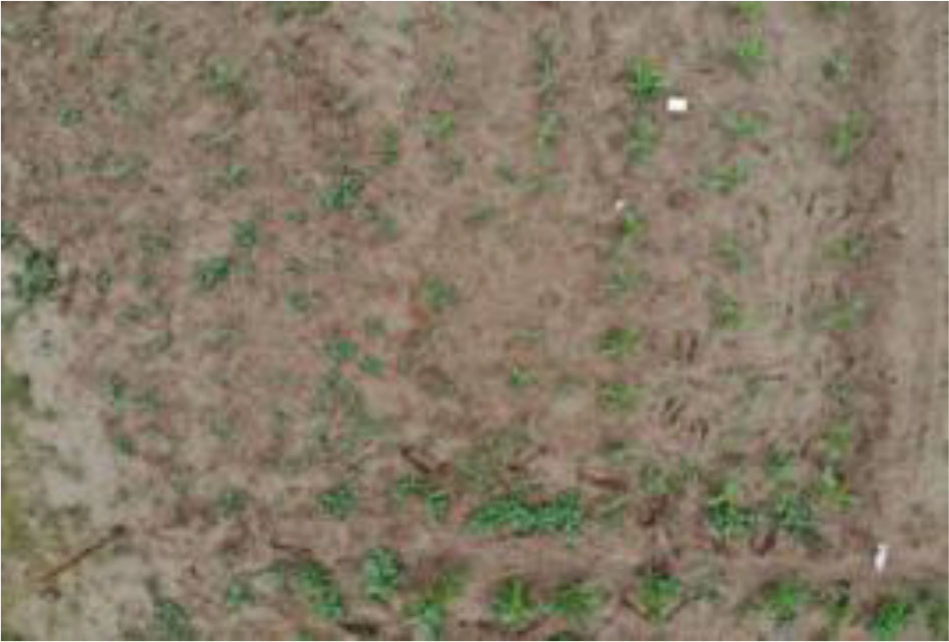
MAVIC AIR	3840×2160 (Video)	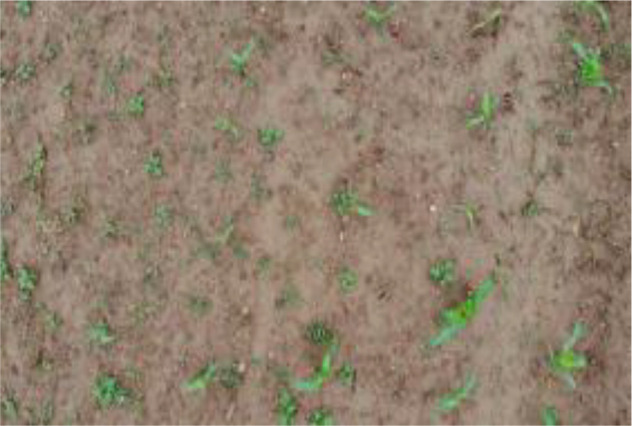	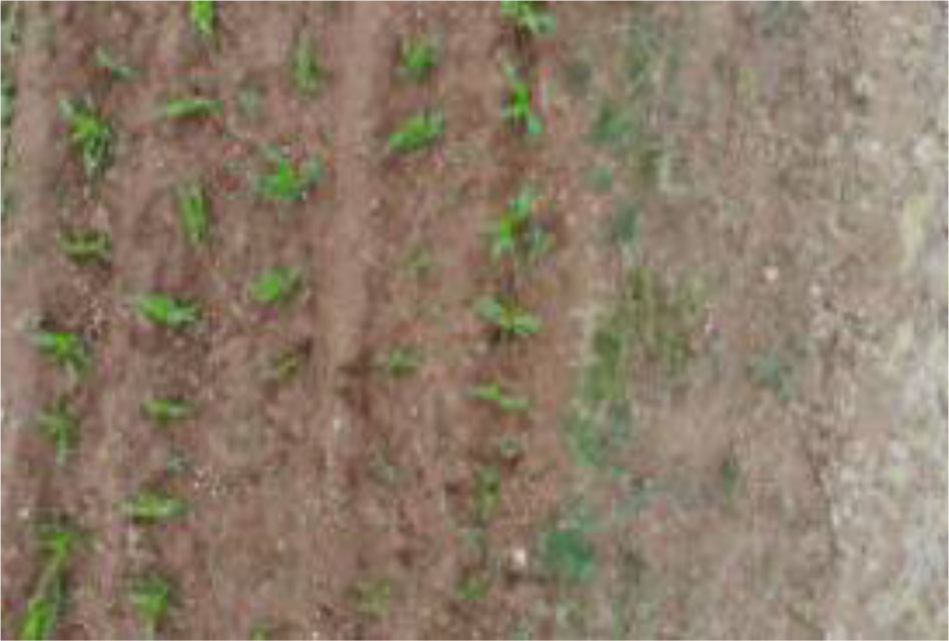	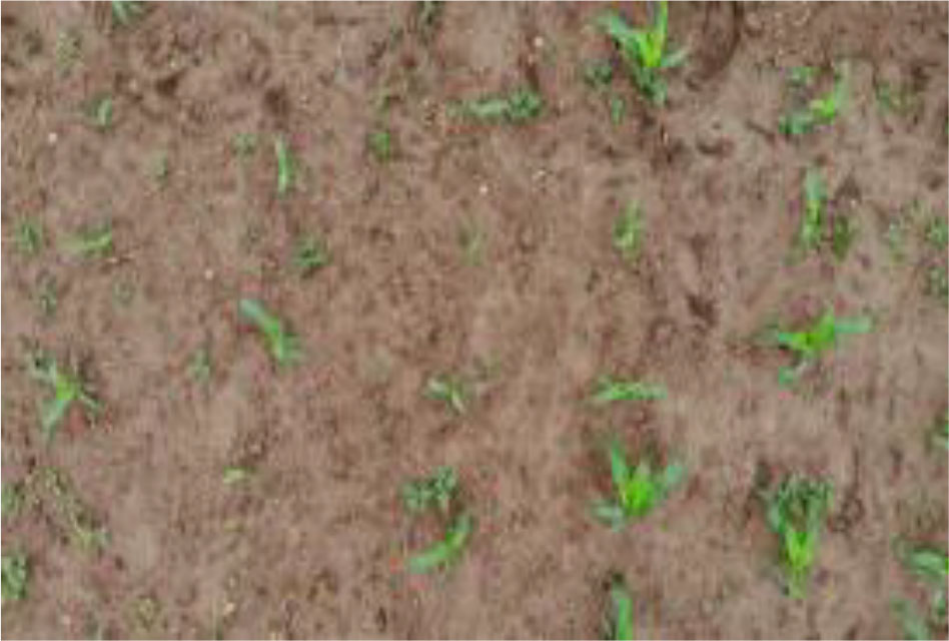

### 2.2 Data preprocessing and dataset generation

**Figure 1:**
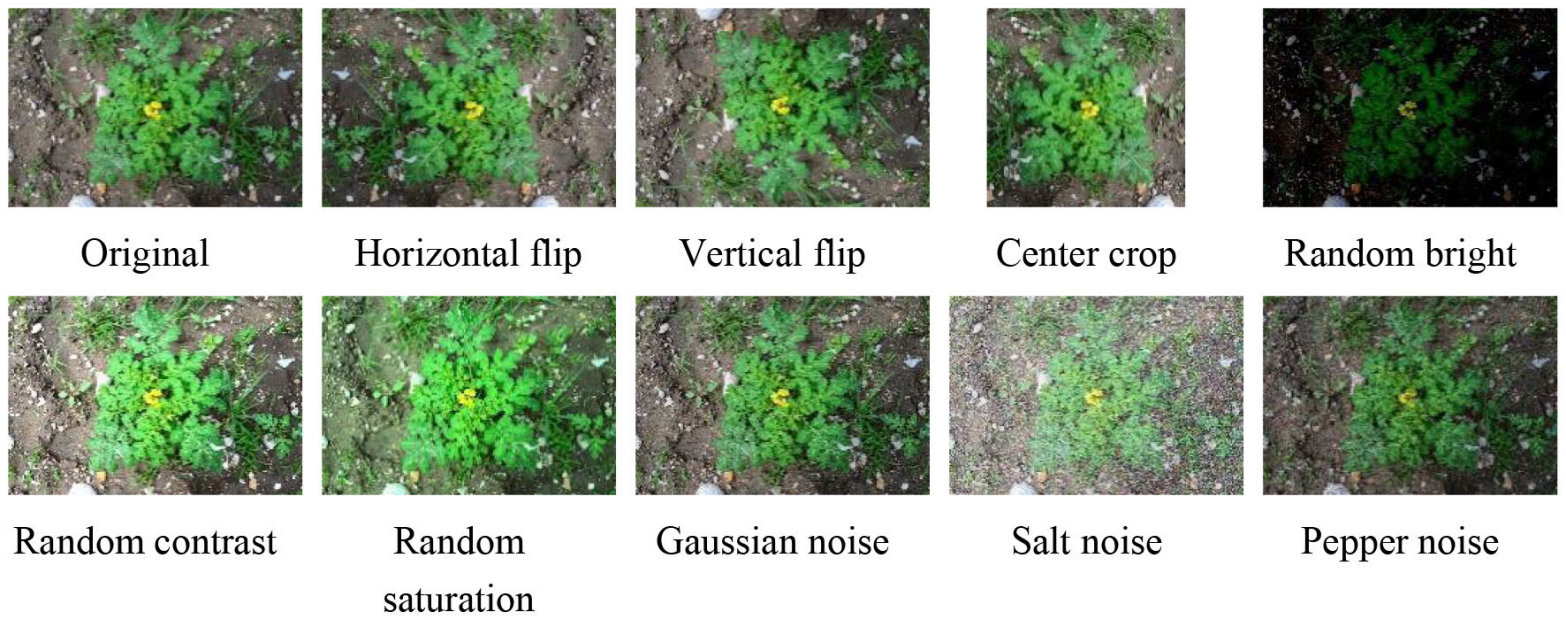
Data augmentation methods and examples.

The pixel proportions of individual plants in different images vary significantly due to differences in equipment, altitude, and the growth stages of SrD. In UAV images, SrD pixels account for roughly 0.1% to 0.6% of the entire image during the seedling and grow thing stages. In contrast, SrD can occupy up to 93.6% of the image in photos captured by cameras during the growing stage. Images are often resized when fed into a deep learning network, typically reduced in size. For instance, YOLO v8 resizes images to 640×640 pixels. To avoid losing detailed information due to image compression during network training, an overlapping image segmentation method was applied to images where the SrD occupies a small pixel area (Wang et al., 2021). This method involves dividing the original images into segments, preserving plant integrity and increasing the number of samples in the dataset, which enhances the model’s generalization and robustness. Additionally, data augmentation techniques were used to enlarge the dataset for images where SrD occupies a large pixel area (**Figure 1**). This processing not only increases the number of images but also helps to balance the dataset, preventing significant bias. After processing, a total of 10,094 images were obtained, containing 51,297 SrD plants.

A target tracking dataset was constructed to train the model’s ability to track the same SrD in a video stream. From the UAV-acquired video sequences at two and three meters, one frame was extracted every ten frames, resulting in a total of 62,664 image pairs with temporal relationships for the dataset.

### 2.3 Deep learning network construction

#### 2.3.1 TrackSolanum deep learning network

In this paper, a TrackSolanum network was designed for the real-time detection, localization, and counting of SrD. It mainly comprises four components: the detection module, tracking module, localization module and counting module. The detection module is primarily based on YOLO_EAND, which quickly and accurately detects SrD plants, providing a reliable database for subsequent processing. The tracking module uses DeepSort to enable multi-object tracking based on the output from the detection module, identifying the same SrD plant across consecutive video frames to avoid repeated identification and counting. The localization module locates the detected SrD plants by searching for their centroids and outputs the specific coordinates of these centroids in each frame, facilitating subsequent removal processes. The counting module prevents repeated counts by invalidating the target ID once it crosses the detection line.

**Figure 2:**
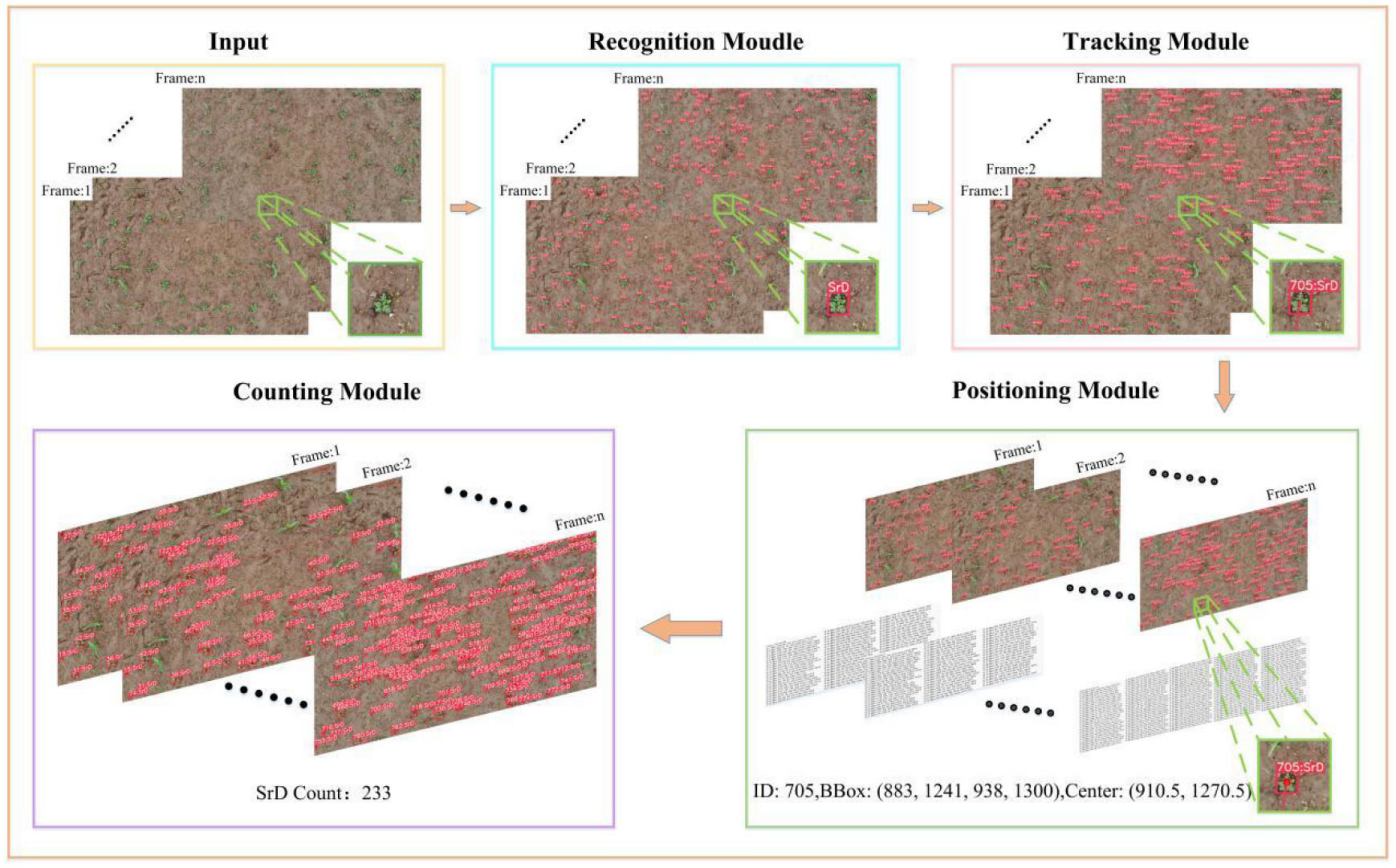
Schematic diagram of the TrackSolanum working process.

When applying the TrackSolanum model, it is loaded onto ground terminal devices, allowing video streams collected by drones or unmanned vehicles to be directly input into the network after transmission. Each frame of the video stream first enters the YOLO_EAND module, which detects the SrD and generates detection boxes. The detection results are then processed by the tracking module, which assigns a unique identity ID to each detected target. These results are input into the localization module, which calculates the centroid coordinates of the detection box as the position of the SrD plant. The counting module counts the number of SrD plants in consecutive video frames based on the identity IDs output by the tracking module. **Figure 2** illustrates the TrackSolanum model’s working process.

#### 2.3.2 Detection module YOLO_EAND

**Figure 3:**
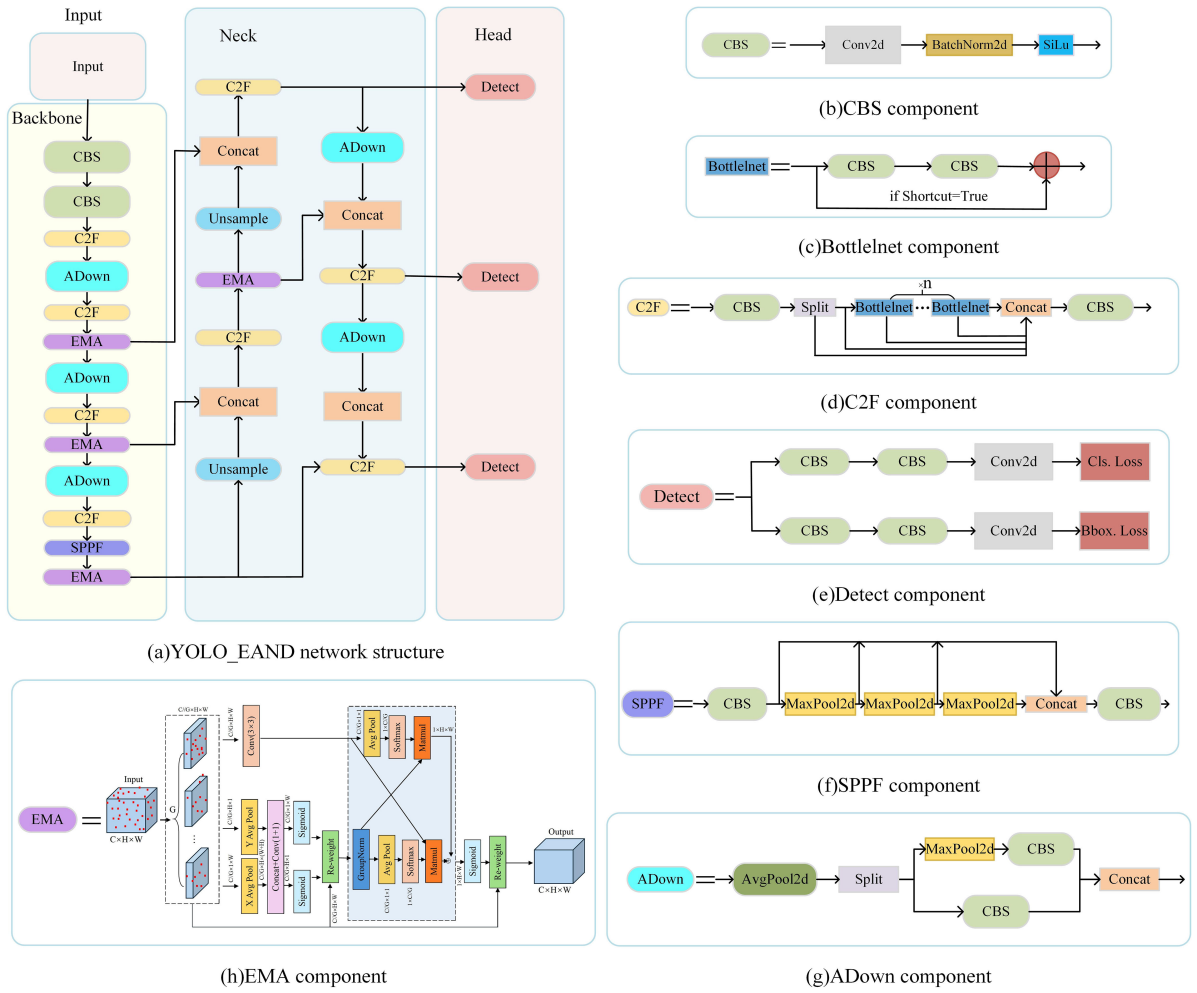
Structure diagram of YOLO_EAND module. **(A)** YOLO_EAND network structure. **(B)** CBS component. **(C)** BottleInet component. **(D)** C2F component. **(E)** Detect component. **(F)** SPPF component. **(G)** ADown component.

YOLO treats the detection task as a regression problem, predicting the bounding box of an object and the corresponding class probabilities directly from the image using a separate neural network (Redmon et al., 2016). In previous work, our team designed a seedling detection network model, YOLO_CBAM, based on YOLO v5 combined with the CBAM attention mechanism. In the current study, we utilized YOLO v8 (Jocher et al, 2023), which offers several advantages over its predecessor, including higher detection accuracy, faster inference, and more efficient utilization of computational resources. These improvements are particularly significant for target detection, especially in real-time tasks where accuracy and speed are crucial. In this paper, we incorporate the EMA (Efficient Multi-Scale Attention) attention mechanism (Ouyang et al., 2023), the ADown downsampling module (Wang et al., 2024), and the NWD (Normalized Wasserstein Distance) loss function (Wang et al., 2021b) into the detection module YOLO_EAND of the TrackSolanum network model. YOLO_EAND consists of three main components: backbone, neck, and head (**Figure 3**). The backbone is responsible for extracting features from the input image, serving as the foundation for subsequent layers in the network for target detection. The neck network, situated between the backbone and the head, performs feature fusion and enhancement. Finally, the head network functions as the decision-making component of the detection model, generating the final detection outcomes.

To enhance the model’s feature extraction capabilities for SrD, YOLO_EAND incorporates the EMA attention mechanism in the Backbone and Neck sections of YOLO v8. This mechanism reorganizes input features into multiple sub-feature groups by reconstructing their channel dimensions, ensuring uniform distribution of spatial semantic features within each group to utilize global information fully. EMA recalibrates channel weights and captures pixel-level pairwise relationships through global information encoding and cross-dimensional interactions within each sub-feature group. This approach preserves critical channel information, reduces computational complexity, and improves model efficiency, as shown in **Figure 3H**. The SrD often appears in complex backgrounds, where its morphological features can be confused with other distracting elements. EMA emphasizes relevant feature channels highlighting SrD-related information and suppressing irrelevant data, enabling more accurate detection in challenging environments (Zhang et al., 2024a). Compared to the CBAM attention mechanism, EMA achieves faster inference speeds while maintaining higher detection accuracy (Xu et al., 2024).

The original YOLO v8 model uses convolution (Conv) for downsampling, which reduces the size of the feature map but can result in the loss of important details, impacting SrD detection performance. To address this, the CBS modules in the Backbone and Neck sections are partially replaced with ADown downsampling modules (**Figure 3G**). The ADown module is an innovative downsampling approach that combines average pooling and max pooling along different processing paths. This combination retains critical feature information while reducing the feature map size, optimizing the number of covariates in the convolutional layer, lowering model complexity, and enhancing detection accuracy and efficiency in resource-constrained environments (Wei et al., 2024; Zhang et al., 2024b). Detecting SrD in real-time tasks often involves small targets, such as newly sprouted seedlings, where feature loss can severely impact model performance. The ADown module’s use of both average and max pooling enriches feature information, enhancing the model’s ability to detect small targets. Additionally, to facilitate system deployment, the ADown module reduces parameters and computational complexity, allowing easier integration into UAVs or other resource-limited platforms.

The original YOLO v8 model utilizes CIOU as its loss function, but traditional IoU metrics are sensitive to positional deviations, especially for small objects, which can degrade detection performance. To address this, a new bounding box measurement method, NWD, is introduced to improve small object detection accuracy. NWD, based on the Wasserstein distance, offers significant advantages over IoU, as it can effectively evaluate similarity even when two frames do not overlap or overlap minimally. This characteristic overcomes IoU’s limitations in small-target detection, especially under conditions of minimal overlap or uncertain target positions, thereby enhancing the model’s adaptability to complex scenes (He and Wan, 2024; Zhang et al., 2024c).

#### 2.3.3 Tracking module DeepSort

When conducting real-time detection of SrD by UAV or UGV, the same plants may appear in consecutive video frames, which can lead to duplicate counts if these instances are not differentiated. This affects the accuracy of the detection. A common approach to address this issue is to set up a detection line in the image and count only the targets that pass through it (Zhang et al., 2023; Li et al., 2024; Ye et al., 2024). However, while this method is effective for counting, it falls short of providing precise localization of detected targets. Accurate localization is essential for target elimination. For instance, each SrD plant must be assigned a unique identifier to distinguish individual plants during herbicide spraying. This ensures that no plant is missed in the video stream and prevents redundant spraying caused by repeated detection. Introducing a tracking module can effectively address these challenges. First, during the movement of the operation platform, the same SrD plant may appear in consecutive frames. If a plant has already been sprayed in a previous frame, the tracking module can associate it with the same plant in subsequent frames, preventing redundant operations. Additionally, the tracking module helps manage intermittent detection due to occlusions or limited viewing angles. For instance, an SrD plant might not be detected in initial frames due to obstructions. However, as the platform moves and the plant becomes visible in later frames, the tracking module can identify it as an untreated target, assign an identifier, and trigger a spraying operation, preventing missed detection. The tracking module also has significant advantages in dynamic environments. For example, during UAV flight, airframe vibrations or wind-induced plant movement can affect detection stability. The tracking module mitigates these detection uncertainties by leveraging temporal continuity. Even when some frames are occluded or fail to detect the plant, the module can use the target’s historical trajectory to link information from previous and subsequent frames, maintaining consistent detection and counting. By incorporating a tracking module, this study overcomes the limitations of static detection methods, improving the accuracy of weed localization and counting. This approach provides reliable technical support for the precise removal of SrD in practical applications.

**Figure 4:**
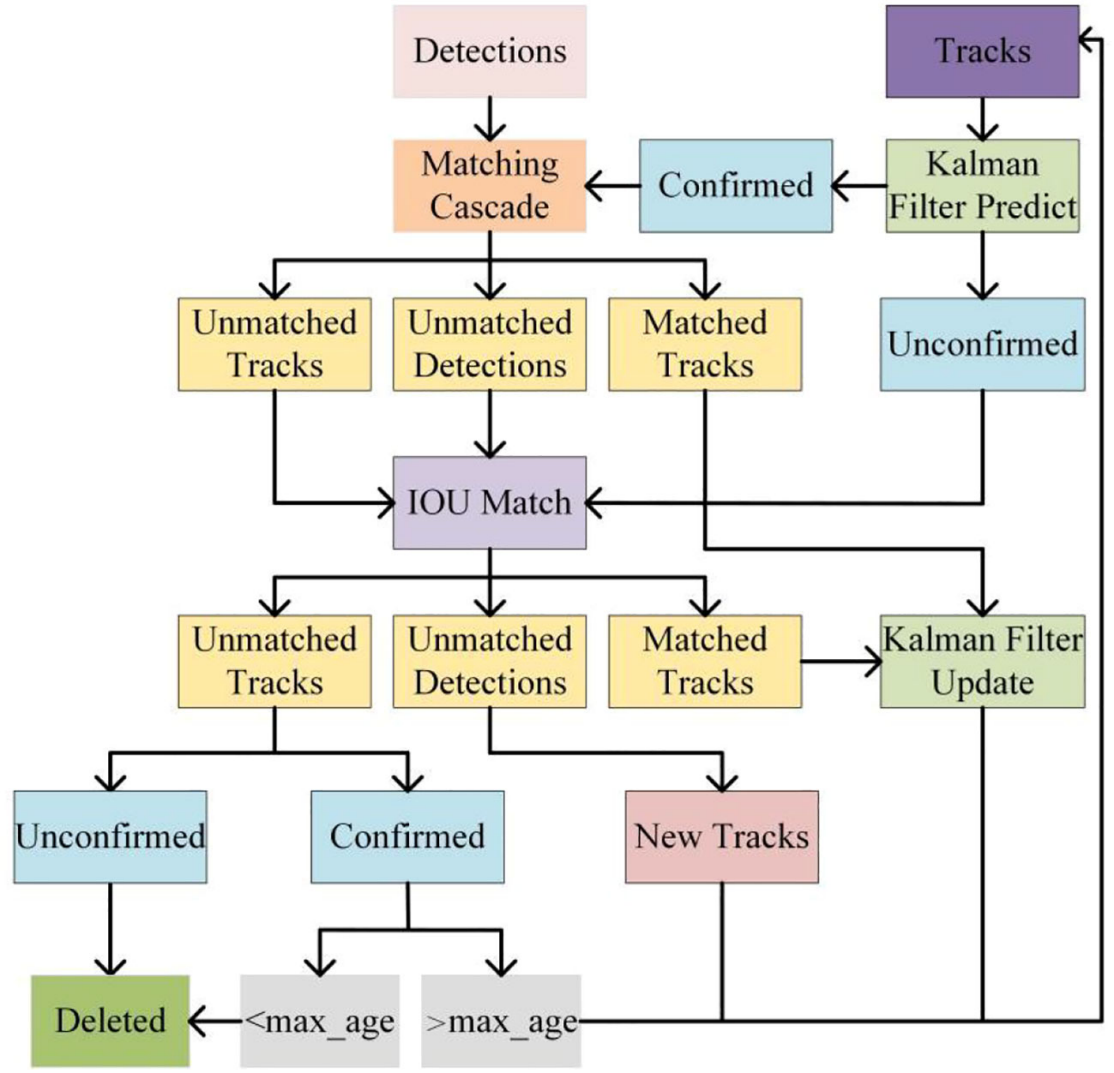
Structure diagram of DeepSort module.

The tracking module, DeepSort, in the TrackSolanum network designed in this paper, tracks the results output by the YOLO_EAND detection module and assigns a unique identity ID to each target. DeepSort is developed based on SORT (Bewley et al., 2016), a simple and efficient method for multi-target tracking. However, SORT’s association metric, which primarily relies on Kalman filtering and the Hungarian algorithm, may struggle in complex scenarios such as overlapping targets and occlusions.To address these issues, DeepSort enhances the SORT algorithm by incorporating cascade matching and new trajectory confirmation, thereby improving the accuracy of target association and reducing the frequency of ID switches. By integrating deep learning-based appearance feature extraction with traditional Kalman filtering and the Hungarian algorithm, DeepSort achieves more robust multi-target tracking, particularly in scenarios involving target overlap, occlusion, and appearance changes. This structure is illustrated in **Figure 4**.

#### 2.3.4 Localization module

Under natural conditions, SrD plants exhibit symmetry, and their leaves are arranged alternate. Previous studies have demonstrated that the geometric center of the smallest enclosing rectangle of a SrD plant closely align with the center of plant itself. The YOLO_EAND detection module outputs a bounding box for each detected SrD plant. Therefore, this study uses the geometric center of the bounding box as the plant’s coordinate position within the image. The calculation for determining these localization coordinates is shown in Equation 1.


(1) $$\left\{ \begin{array}{c} x_{0}=(x_{1}+x_{2})/2\\ y_{0}=(y_{1}+y_{2})/2 \end{array}\right.$$

Where, *x*_0_ and *y*_0_ represent the horizontal and vertical coordinates of the geometric center of the bounding box, respectively. *x*_1_ and *y*_1_ are the horizontal and vertical coordinates of the upper left corner of the bounding box, while *x*_2_ and *y*_2_ indicate the horizontal and vertical coordinates of the lower right corner.

**Figure 5:**
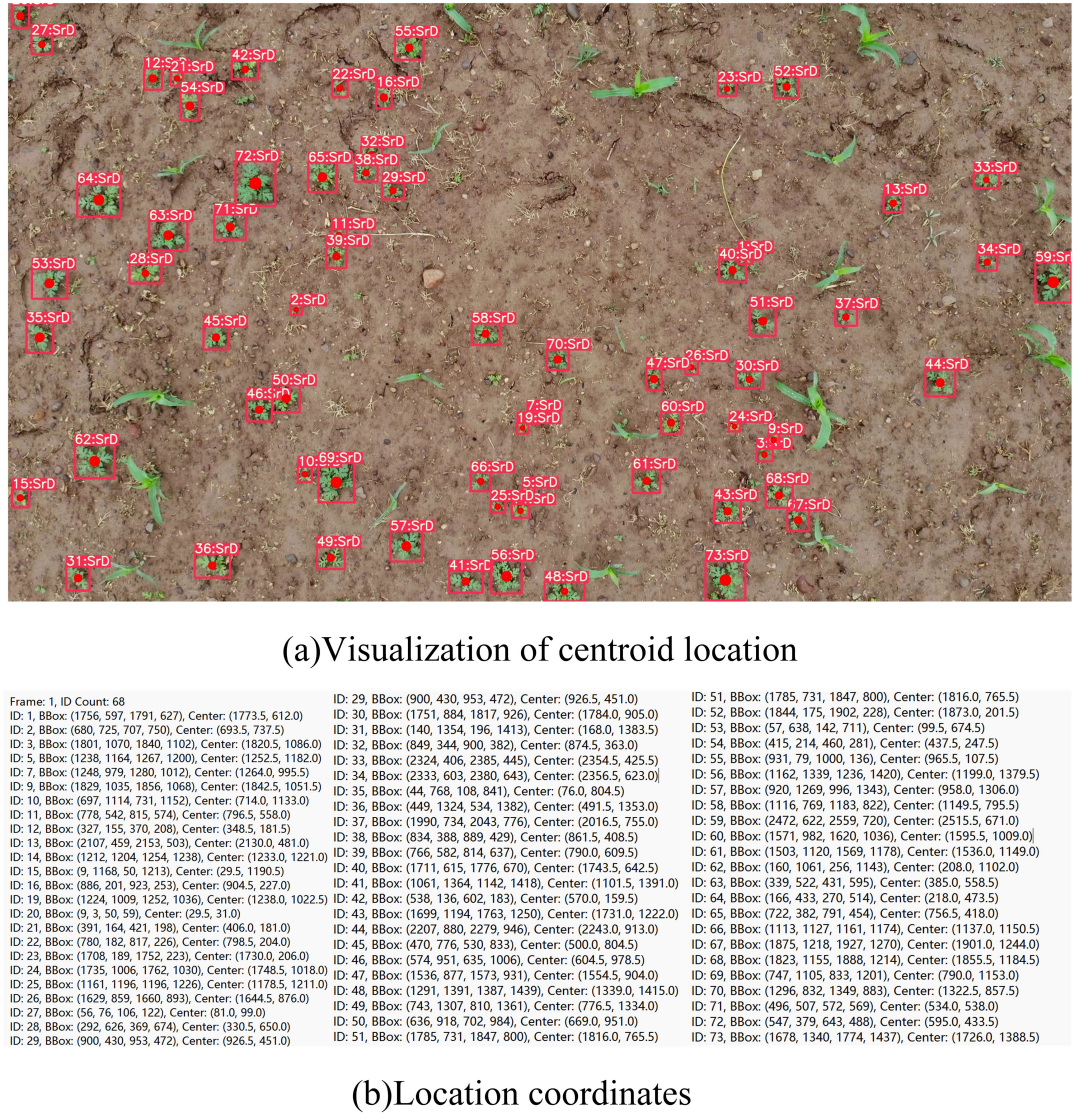
Localization examples of SrD.

After the video is input into the TrackSolanum network, the YOLO_EAND module processes each frame, generating detection boxes for the identified SrD plants and calculating the coordinates of their center points. A txt file is then created to log the identity IDs, detection box coordinates, and center point coordinates of all detected SrD in each frame. The primary goal of localizing SrD is to facilitate subsequent real-time removal. Using the centroid of the detection box for localization enables quick positioning of each plant, minimizing responses time for future real-time clearing operations. The localization results are shown in **Figure 5**. In **Figure 5A**, the visualization of centroid localization is presented, with the centroids of the detection boxes marked by solid red dots, indicating the precise position of the plants. **Figure 5B** shows the coordinate information for each SrD, as recorded in the txt file, which provides critical data for subsequent statistical analysis.

#### 2.3.5 Counting module

Counting the number of SrD plants in a specific region allows for the quantification of their density and distribution, providing an accurate assessment of their impact and essential data for developing targeted control measures. Precise counting results enable agricultural departments and related organizations to allocate resources more effectively and implement more efficient eradication or control strategies in high-density invasion areas, reducing the adverse effects of Solanum rostratum on ecosystems and agriculture. Additionally, counting facilitates the prediction of the SrD’s spread patterns and trends.

For each frame in the video, the YOLO_EAND detection module identifies SrD plants, while the DeepSort tracking module assigns a unique identity ID to each detected plant. Ideally, the identity ID of the same SrD plant should remain consistent across consecutive video frames. However, changes in perspective during detection and tracking can sometimes cause the same target to be identified as a different object in adjacent frames, leading to the assignment of new IDs. Calculating the total number of SrD directly from these IDs could result in inaccurate counts. To address this issue, we developed a counting method where the target ID becomes invalid once the SrD crosses a designated detection line.

This program integrates the coordinated functions of target detection, tracking and counting to enhance counting accuracy. In this approach, a detection line is set within the program as a trigger for counting. When the lower boundary of the detection box (i.e., the vertical coordinate *y*_2_) is greater than the y-coordinate of the detection line, and the y-coordinate of the detection line is less than the upper boundary of the detection box (i.e., the vertical coordinate *y*_1_), the counting condition is triggered, indicating that the detection box for SrD has crossed the detection line. Once this condition is met, the system checks whether the unique ID of the current detection box has been previously counted. If the ID has not yet been counted, the system increments the count by one and saves the ID in a list named Count_ids to prevent duplicate counts. If the ID has already been counted, the system continue to monitor and track the SrD to ensure consistent observation. Finally, the counting results are output to the detected video for further analysis and evaluation.The total number of SrD in the video can be calculated using Equation 2.


(2) $$Z=\sum_{i=0}^{n}C_{\text{i}}$$

Where, *Z* represents the cumulative number of SrD, *C*_i_ denotes the number of SrD that passed through the detection line in frame *i*, and *n* is the total number of frames in the video.

**Figure 6:**
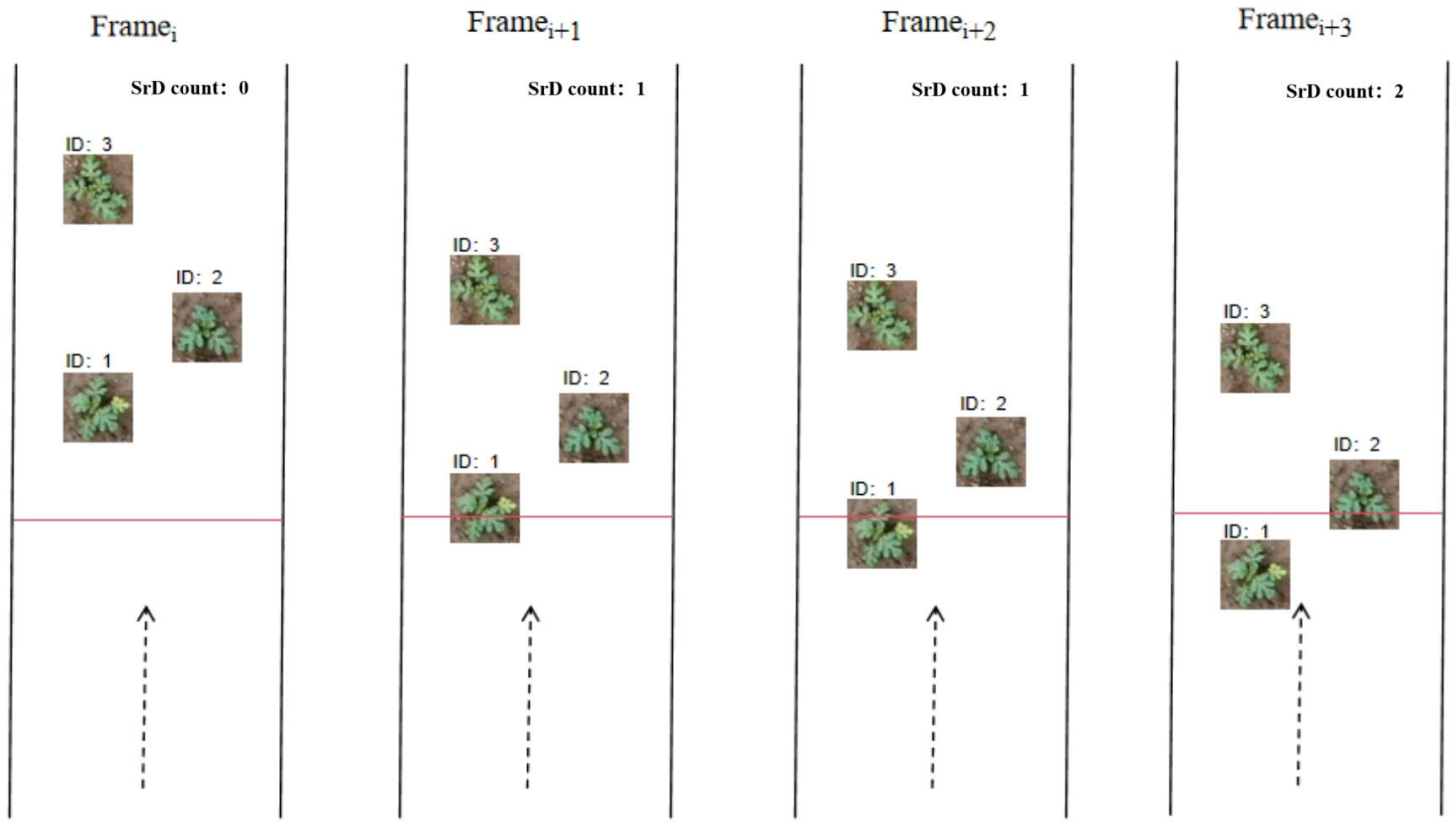
Schematic diagram for calculating the number of SrD plants.

A schematic diagram of the counting method for the ID over-the-line failure counting method is shown in **Figure 6**. This method effectively reduces counting errors caused by changes in ID and mitigates the impact of variations in the speed of UAV or ground vehicles, which can lead to the detection box of the same target repeatedly crossing the counting line in consecutive video frames. As a result, it enhances the counting accuracy and reliability of SrD.

### 2.4 Network model hyperparameter setting and operating environment

In this paper, the deep learning model TrackSolanum is constructed using the PyTorch 2.1.2 framework, with the program code written in Python. The model operates on a desktop computer equipped with a 12th Gen Intel^®^ Core™ i9-12900K CPU, an NVIDIA GeForce RTX 4090 GPU, CUDA version 11.8, and the Microsoft Windows 11 operating system.

The batch size was set to thirty-two during model training, and the probability of performing Mosaic in the image enhancement part was set to ten. The Stochastic Gradient Descent (SGD) optimizer was employed, with the maximum iteration period (Epoch) set to 300. The initial learning rate was set to 0.01, the final learning rate to 0.0001, the momentum of the optimizer to 0.937, the weight decay coefficient to 0.0005, the image mosaic probability to 1.0, the image panning ratio to 0.1, the image scaling ratio to 0.5, and the image left-right flip ratio to 0.5. To ensure fairness and comparability of model performance in the experiments, no pre-trained weights were used during model training. The training input image size was set to 640×640. The total dataset comprises 10,094 image pairs, which are randomly partitioned in an 8:1:1 ratio. The training set includes 8,077 images, the validation set comprises 1,010 images, and the test set contains 1,007 images.

The tracking module DeepSort has the maximum iteration period (Epoch) set to one hundred, the learning rate to 0.1, and the batch size (Batch_size) set to sixty-four for model training. The min_confidence is set to 0.2, max_iou_distance is set to 0.3, and nms_max_overlap is set to 0.5.The dataset for target tracking comprises a total of 62,664 pairs of images, which are randomly divided using a ratio of 8:1:1, where the training set contains 50,127 pairs of consecutive images with temporal relationships, the validation set contains 6,265 pairs of consecutive images with temporal relationships, and the test set contains 6,272 pairs of consecutive images with temporal relationships.

### 2.5 Network model performance evaluation metrics

#### 2.5.1 Evaluation metrics for the YOLO_EAND detection module

In this paper, precision (*P*), recall (*R*), average precision (*AP*), and frames per second (*FPS*) are used as evaluation metrics to assess the performance of the YOLO_EAND module. *P* quantifies the ratio of correctly identified SrD (true positives) to the total number of instances classified as SrD by the model. *R* is the proportion of SrD instances correctly identified by the model to the total number of SrD. *AP* is the average precision across different recall rates and can also be viewed as the area under the precision-recall curve. *AP* is a crucial evaluation metric for object detection algorithms, and a higher *AP* value indicates better detection performance of the model on the given dataset. Detection speed can be evaluated by *FPS*, which represents the number of images that can be processed per second. *P*, *R*, *AP*, and *FPS* can be calculated using Equation 3.


(3) $$\left\{ \begin{array}{c} P=\frac{TP}{FP+TP}\\ R=\frac{TP}{FN+TP}\\ \begin{array}{c} AP=\sum \int_{0}^{1}P(R)dR\\ FPS=\frac{1}{t} \end{array} \end{array}\right.$$

Where, *TP* denotes the number of SrD plants correctly identified by the network model, while *FP* indicates the number incorrectly detected. *FN* refers to the SrD plants that the model failed to detect. The variable *t* signifies the time required to process a single image, measured in seconds.

#### 2.5.2 Evaluation metrics for the DeepSort tracking module

The performance of the tracking module DeepSort can be evaluated using Identification Switch (*IDSW*), Multiple Object Tracking Accuracy (*MOTA*) and Identification F1 (*IDF1*) (Rong et al., 2023; Villacres et al., 2023). *IDSW* refers to the number of ID switches of SrD during video tracking. It counts the occurrences of incorrect target ID switches throughout the video sequence, revealing whether the algorithm can correctly maintain target identity during occlusion, disappearance, or reappearance. A lower *IDSW* value indicating better performance. *MOTA*, recognized as a standard metric for assessing the efficacy of multi-object tracking systems, accounts for three key types of tracking errors: false positives, false negatives, and identity switches. *MOTA* provides an overall evaluation of the tracking algorithm’s performance through a single metric, allowing direct comparison between different algorithms. A higher *MOTA* score, nearing the value of one, denotes superior tracking performance. *IDF1* is an important metric for evaluating the consistency of identity assignments in multi-object tracking scenarios. *IDF1* represents the proportion of detected and tracked targets assigned the correct ID. It is a comprehensive metric that evaluates correctly detected targets, false positives, and missed detections, similar to the traditional F1 Score. By using the harmonic mean, IDF1 balances precision and recall. The performance of DeepSort in weed detection and tracking can be comprehensively evaluated using the three metrics mentioned above. These metrics effectively reflect the model’s robustness in handling complex scenarios. Considering these evaluation metrics together helps provide a holistic understanding of DeepSort’s strengths and weaknesses in detecting SrD.

It represents the proportion of detected and tracked objects that are assigned the correct ID. A value of *IDF1* closer to one indicates higher precision in tracking specific targets. *MOTA* and *IDF1* can be calculated using Equation 4.


(4) $$\left\{ \begin{array}{c} MOTA=1-\frac{\sum_{\text{t}}(FN_{t}+FP_{t}+IDSW_{t})}{\sum_{t}GT_{t}}\\ IDF1=\frac{2IDTP}{2IDTP+IDFN+IDFP} \end{array}\right.$$

Where, *t* represents the index number of the current video frame. *FP_t_* denotes the number of SrD misidentified as other objects in the *t*-th frame, *FN_t_* denotes the number of SrD not detected in the *t*-th frame. *GT_t_* represents the total number of SrD detected in the *t*-th frame, and *IDSW_t_* denotes the number of ID switches of SrD in the *t*-th frame. *IDTP* represents the number of SrD correctly tracked throughout the video, *IDFP* represents the number of SrD incorrectly tracked, and *IDFN* represents the number of SrD lost during the tracking process.

#### 2.5.3 Counting accuracy evaluation metrics

The counting performance of SrD can be evaluated using the error rate (*ER*). The *ER* can be calculated using Equation 5.


(5) $$ER=\frac{\left|Count-Ground\;Truth\right|}{Ground\;Truth}$$

Where, Count represents the number of SrD detected by the model in the video, and Ground Truth represents the manual count of SrD in the video.

### 2.6 Field test

To verify the effectiveness of the TrackSolanum network model designed in this paper for practical applications, field tests were conducted on June 23, 2024, and July 30, 2024, at the invasion sites of SrD in Zhangjiakou City, Hebei Province, specifically at MP 5# (40.46.57N, 114.42.4E) and MP 6# (40.51.5N, 114.54.20E). The test site featured SrD in a natural state of growth, and video data were collected using a MAVIC AIR. The video captured by the drone had a resolution of 3840 × 2160 and a frame rate of 30 frames per second. The camera was kept perpendicular to the ground while the drone was in flight. At the MP 5#, flight heights of 2 and 3 meters were used; at the MP 6#, heights of 2, 3, 4 and 5 meters were used. Throughout the field tests, the drone flew at a speed of 1 m/s along a straight line at a constant speed, ensuring no overlap in the flight paths.

## 3 Results

### 3.1 Detection results of the test set

The 1,007 images in the test set were fed into the trained TrackSolanum network model, and the detection results from the YOLO_EAND module are presented in **Table 2**. The test set contained a total of 5,871 SrD plants. The model detected 5,543 plants, indicating that it accurately identified the weed in most cases. However, there were 308 instances of detection errors, where the model incorrectly identified some objects that were not SrD as targets. Further analysis of these false positive samples revealed that other crops and weeds in the detection images were misidentified as SrD due to morphological similarities. These errors not only increased the counting inaccuracies but could also affect the model’s practical application in complex environments. The model missed 328 instances of SrD, with false negatives primarily occurring when the plants were obscured, small, or had indistinct features, affecting detection comprehensiveness. Although the YOLO_EAND model incorporated the EMA, ADown downsampling, and NWD loss function to enhance feature extraction and small target detection capabilities, the analysis indicates that it still exhibits certain errors under complex background conditions and specific scenarios.

**Table 2:** YOLO_EAND identification results on the test set.

Actual number	*TP*	*FP*	*FN*	*AP*	*P*	*R*	Time consume	*FPS*
5,871	5,235	308	328	0.981	0.947	0.944	80.326	98

**Figure 7:**
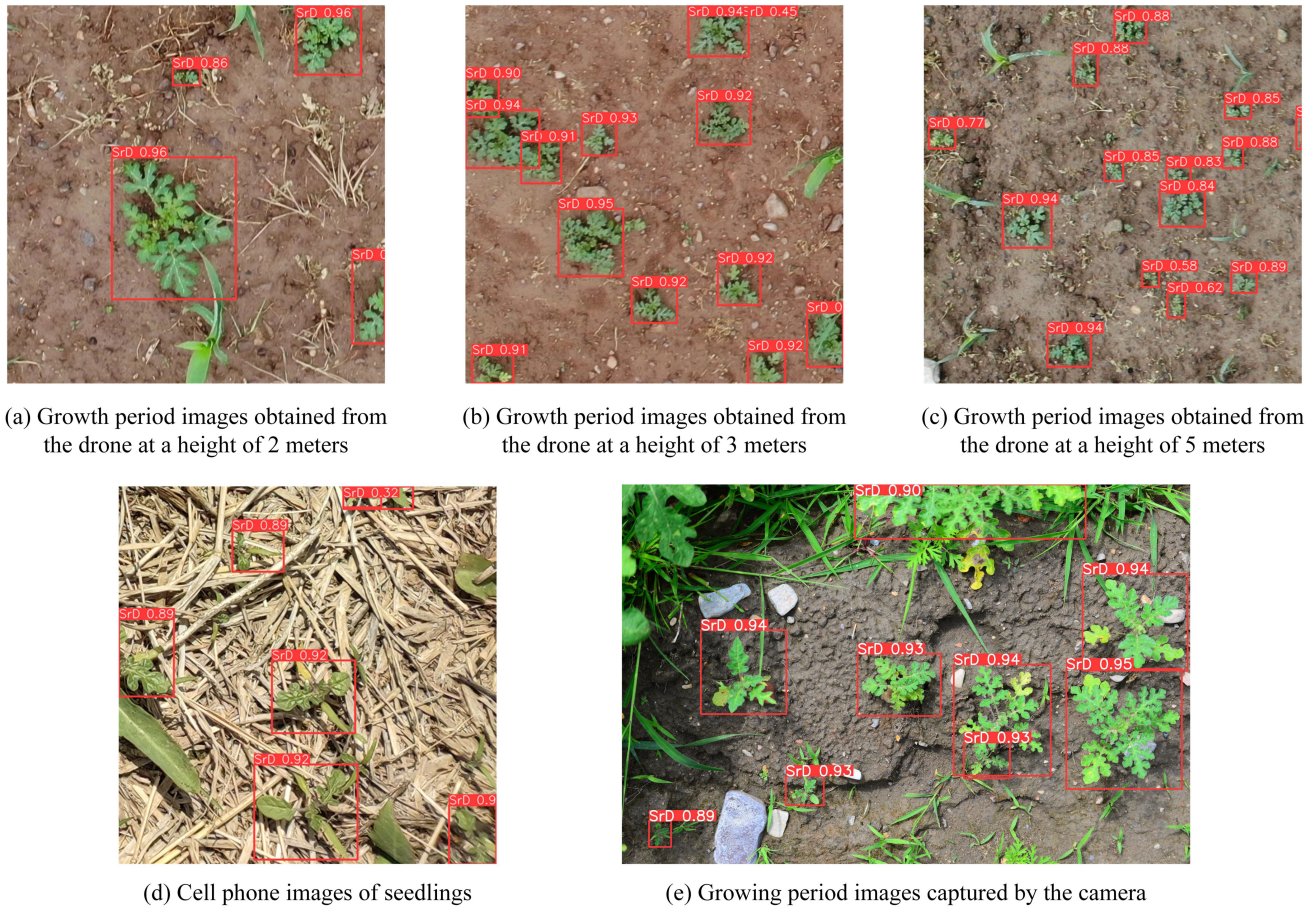
Partial test set detection results. **(A)** Growth period images obtained from the drone at a height of 2 meters. **(B)** Growth period images obtained from the drone at a height of 3 meters. **(C)** Growth period images obtained from the drone at a height of 5 meters. **(D)** Cell phone images of seedlings. **(E)** Growth period images captured by the camera.

The *P*, *R* and *AP* of the YOLO_EAND model are 0.947, 0.944 and 0.981 respectively. The model processed 1,007 images in a total time of 80.326 seconds, with an *FPS* of 98. **Figure 7** shows the detection results for some images from the test set. It can be observed that the TrackSolanum network applicable to images captured by different devices and is capable of detecting SrD plants at various growth stages.

### 3.2 Field test results

At MP 5#, most of the SrD plants were in the growth stage with more than six leaves, although a few seedlings had fewer than six leaves. Four video segments were captured at heights of 2 and 3 meters, respectively. The videos captured at 2 meters had a total duration of 106 seconds, while those at 3 meters totaled 95 seconds. **Table 3** presents the test results of the field videos.

**Table 3:** Experimental results for videos at different flight altitudes at MP 5#.

Video	Flight altitude	Time	*P*	*R*	*MOTA*	*IDF1*	*IDSW*	Ground truth	Model count	*ER*/%	*FPS*
1	2m	26s	0.930	0.936	0.749	0.934	31	229	222	3.057	21
2	2m	25s	0.941	0.976	0.867	0.958	27	506	497	1.779	19
3	2m	21s	0.975	0.981	0.833	0.976	33	246	236	4.065	15
4	2m	34s	0.955	0.985	0.861	0.972	43	471	467	0.849	13
	average	26.5s	0.950	0.970	0.828	0.960	34	363	356	2.438	17
5	3m	21s	0.887	0.921	0.717	0.907	13	70	66	5.714	40
6	3m	50s	0.806	0.961	0.646	0.859	14	173	169	2.310	26
7	3m	14s	0.860	0.914	0.765	0.889	6	71	75	5.634	56
8	3m	10s	0.829	0.940	0.702	0.897	5	41	39	4.878	57
	average	23.75s	0.846	0.934	0.708	0.888	10	89	87	4.634	45

At a height of 2 meters, the *P* and *R* for detecting SrD reached 0.950 and 0.970, respectively. The tracking performance metrics *MOTA* and *IDF1* in consecutive video frames reached 0.828 and 0.960, with an *IDSW* of 34 and an *ER* of 2.438%. The *FPS* was 17. Although the model performs excellently in detection and tracking, the need to save the location information of SrD in real-time to a specified txt file during the processing of each frame results in a decrease in video processing speed compared to single-frame image processing. This additional data storage operation may cause a delay when processing a large number of video frames, but this delay mainly occurs during the data storage phase and does not have an impact on the real-time nature of data acquisition during UAV flight. In this system architecture, video data is transmitted to the ground terminal devices for processing via the UAV, allowing detection results to be output quickly after input into the model, ensuring that the real-time of the field operation is not affected.

At a height of 3 meters, the *P* and *R* for detecting SrD reached 0.846 and 0.934, respectively. The tracking performance metrics *MOTA* and *IDF1* in consecutive video frames were 0.708 and 0.888, with an *IDSW* of 10 and an *ER* of 4.634%. The *FPS* was 45. Although the *FPS* is higher and processing faster at a 3-meter altitude, the performance at 2 meters is superior in terms of detection, tracking and counting accuracy. Overall, the TrackSolanum model efficiently accomplishes the detection, localization, and counting tasks for SrD at various flight heights. While the processing speed is slightly reduced due to the need to save detection results for each frame, this does not affect the real-time application of the drone. The data processing delay primarily occurs on the ground terminal devices; once data analysis is completed, the system can quickly output detection results. Therefore, the TrackSolanum model ensures real-time data collection and transmission during drone flight, making it suitable for on-site applications.

At the MP 6#, the plants were in a growth stage with more than six leaves. **Table 4** presents the test results from the field video. At 2 and 3 meters flight altitude, the model showed better detection performance, with *P* values of 0.925 and 0.941, and *R* values of 0.943 and 0.946, respectively, indicating that the model accurately identified most targets with a low false detection rate. As the flight altitude increased, detection performance declined, particularly at 5 meters where *P* dropped to 0.774, and *R* also decreased. This suggests that higher altitudes may result in some targets being inaccurately detected or missed, though overall accuracy remained within acceptable limits. For the *MOTA*, the 2 and 3 meter altitudes performed better with scores of 0.819 and 0.830, respectively, indicating effective target tracking at these altitudes. However, *MOTA* decreased significantly at 4 and 5 meters, likely due to factors such as reduced resolution and motion blur. The *IDF1* value peaked at 0.915 at at 3 meters, indicating optimal tracking quality at this altitude, but gradually decreased with increasing height, reaching 0.740 at 5 meters. *IDSW* was lower at 2 and 3 meters, with 38 and 32 switches, indicating more stable tracking, while at 4 and 5 meters, *IDS*W increased to 43 and 57, suggesting a greater tendency for ID mis-switching. Counting results indicated *ER* of 5.116% and 4.859% at 2 and 3 meters, respectively, showing good detection performance at these altitudes. As flight altitude increased, counting errors rose significantly, with the *ER* reaching 10.803% at 5 meters, indicating a greater likelihood of missed detections at higher altitude, impacting counting accuracy. Due to the relatively sparse density of SrD in this plot, the amount of positional information processed per frame was low, leading to a notable increase in detection speed. Detection speed varied with flight height, being highest at 2 meters (reaching 83 *FPS*), and decreasing to 36 *FPS* at 4 meters. At 5 meters, detection speed showed a slight improvement, likely because fewer targets were detected at this height, reducing the positional information needed per frame and leading to an increase.

**Table 4:** Field test results at MP 6#.

Video	Flight altitude	Time	*P*	*R*	*MOTA*	*IDF1*	*IDSW*	Ground truth	Model count	*ER*/%	*FPS*
9	2m	75s	0.925	0.943	0.819	0.910	38	215	204	5.116	83
10	3m	60s	0.941	0.946	0.830	0.915	32	247	235	4.859	79
11	4m	53s	0.885	0.910	0.743	0.874	43	315	294	6.667	36
12	5m	43s	0.774	0.827	0.631	0.740	57	361	322	10.803	42

## 4 Discussion

### 4.1 Performance analysis of trackSolanum network in detecting SrD

To validate the effectiveness of the YOLO_EAND module within the proposed TrackSolanum network model, an ablation experiment was conducted. This experiment assessed the performance impact of the EMA, ADown downsampling, and the NWD loss function on the YOLO v8 detection network under different configurations, using the same training and testing sets for comparison. **Table 5** displays the ablation test results. Incorporating the EMA led to significant improvements in *P*, *R*, *AP*, *TP* and *FPS*, while reducing the numbers of *FP* and *FN.* This indicates that the EMA enhances the model’s feature extraction capability, allowing for more accurate SrD detection in complex backgrounds or occluded situations. It improves detection accuracy and reduces false positives, aiding in the precise extraction of target centroids for the localization module. While edge detail detection enhances accuracy, real-world large-scale applications often prioritize overall model performance and real-time capabilities. The ADown downsampling module effectively reduces model parameters, decreasing the model size from 6.3MB to 5.5MB, and significantly cuts *FP*, thereby improving model accuracy. This demonstrates that ADown maintains crucial semantic information while lowering the spatial dimension of the feature map, optimizing detection efficiency. Compared to YOLO v8n, the ADown module results in fewer false positives and better background handling, essential for processing high-resolution images with complex scenes during UAV operations, enhancing system adaptability. The addition of the NWD loss function improves all performance metrics, particularly *FPS*, which rises from 72 to 102, greatly enhancing real-time video processing capabilities. This boost ensures that the model is suitable for large-scale field environments, providing efficient detection while maintaining high accuracy. Compared to the original YOLO v8n network, YOLO_EAND raises *P* by 2.5 percentage points, *R* by 1.8 percentage points, *AP* by 0.9 percentage points, and increases *FPS* from 72 to 98, achieving a 12.698% model reduction.

**Table 5:** Results of ablation experiments.

Model	Model size	*TP*	*FP*	*FN*	*P*	*R*	*AP*	*FPS*
YOLOv8n	6.3MB	5439	458	432	0.922	0.926	0.972	72
YOLOv8n+EMA	6.3MB	5496	369	375	0.937	0.936	0.978	91
YOLOv8n+ADown	5.5MB	5437	285	434	0.950	0.926	0.979	70
YOLOv8n+NWD	6.3MB	5478	311	393	0.946	0.933	0.978	102
YOLOv8n+EMA+ADown	5.5MB	5448	279	423	0.951	0.928	0.978	91
YOLOv8n+EMA+NWD	6.3MB	5532	396	339	0.933	0.942	0.979	89
YOLOv8n+ADown+NWD	5.5MB	5495	330	376	0.943	0.936	0.978	94
YOLO_EAND	5.5MB	5543	308	328	0.947	0.944	0.981	98

Our team previously employed the DeepSolanum network to detect and segment established populations of SrD and calculated coverage. Although effective detection was achieved at higher flight altitudes (15 meters), it depended on the plants reaching a certain growth stage and forming a population. In drone images, SrD tends to appear in clusters or groups, making individual plant identification and counting impossible. This suggests that SrD has begun to negative impact the local ecosystem. Therefore, to detect SrD invasion at an early stage, our team developed a YOLO_CBAM model based on YOLO v5 for the early recognition of individual plants.

To further improve the identification accuracy of SrD, this paper enhances the recognition model using YOLO v8 as the foundational network. It incorporates the EMA, ADown downsampling, and the NWD loss function to construct the YOLO_EAND module within the TrackSolanum model for detecting SrD. **Table 6** presents a comparison of the recognition results of YOLO_EAND with the SSD and YOLO_CBAM models under the same training and testing datasets. As shown in **Table 7**, YOLO_EAND is the lightest model, with a memory footprint of 5.5 MB, demonstrating significant advantages in storage and computational efficiency compared to other models. When compared to SSD, YOLO_EAND exhibits substantial improvements across all performance metrics, with *TP* increasing from 5,109 to 5,543, indicating better performance in accurately detecting targets. *FP* decreased by 318, and *FN* were reduced by 434, highlighting a significant enhancement in reducing misdetections and omissions. *P*, *R*, and *AP* improved by 5.6 percentage points, 7.4 percentage points, and 8.0 percentage points, respectively. Additionally, the inference speed of YOLO_EAND is 2.579 times that of SSD, showcasing its strong real-time processing capability while being only 6.071% of the size of SSD. Compared to YOLO_CBAM, although YOLO_EAND shows a slight decline in *TP* and *R*, along with an increase in *FN*, it outperforms in model size, *FP*, *P*, and *AP*, with a more notable enhancement in *FPS*, reaching 1.849 times that of YOLO_CBAM. Furthermore, the size of the YOLO_EAND model is only 36.913% that of YOLO_CBAM, making it more suitable for deployment on edge devices for on-site detection.

**Table 6:** Results of comparative experiments.

Model	Model size	*TP*	*FP*	*FN*	*P*	*R*	*AP*	*FPS*
SSD	90.6MB	5109	626	762	0.891	0.870	0.901	38
YOLO_CBAM	14.9MB	5614	427	257	0.929	0.956	0.954	53
YOLO_EAND	5.5MB	5543	308	328	0.947	0.944	0.981	98

**Table 7:** Detection results at different growth stages.

Dataset	Number of images	*P*	*R*	*AP*	*FPS*
Seedling stage	230	0.954	0.986	0.990	120
Growth Stage	183	0.950	0.967	0.972	87

### 4.2 The impact of different growth stages of SrD on detection performance

**Figure 8:**
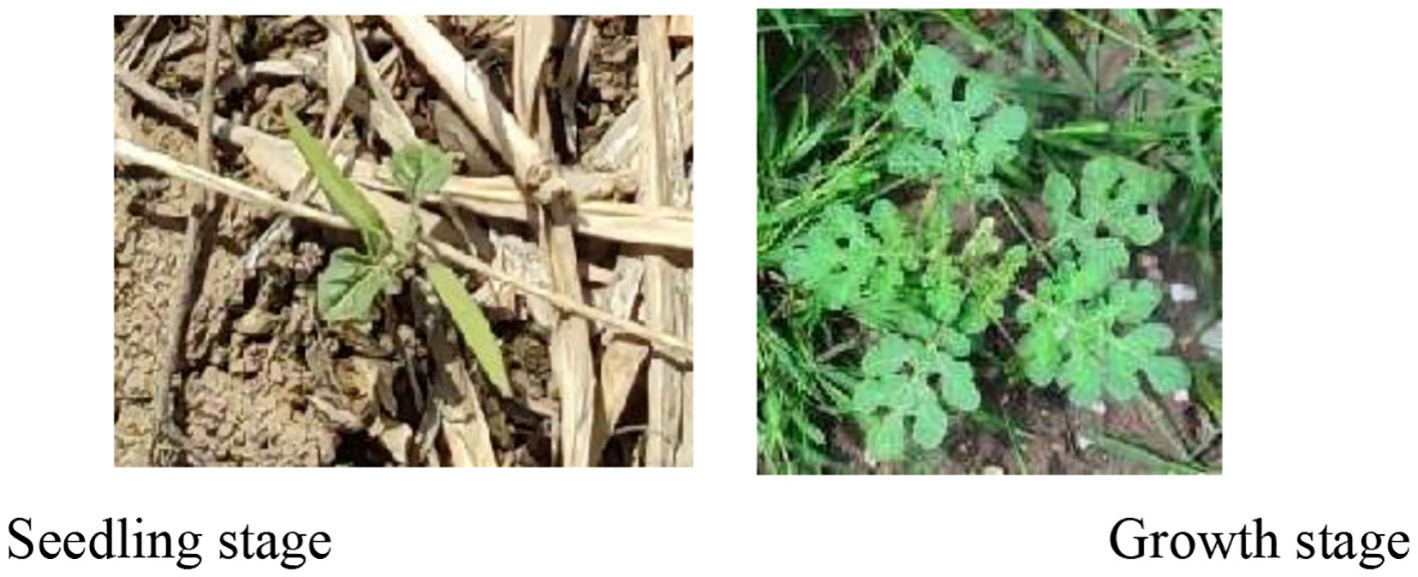
Seedling and growth stages of SrD.

The leaf shape of SrD varies at different growth stages. During the seedling stage, the plant features two lanceolate cotyledons, which transform into pinnate leaves during the growth stage (**Figure 8**). Although the TrackSolanum network model was trained on a mixed dataset without distinguishing between the seedling and growth stages, further analysis is required to assess its performance when testing the SrD at these different stages. To this end, the test set was divided into two subsets representing the seedling and growth stages, with each subset undergoing separate testing. **Table 7** presents the test results, indicating that the TrackSolanum model achieved *P*, *R*, *AP*, and *FPS* scores of 0.954, 0.986, 0.990, and 120, respectively, for the SrD seedling test set. For plants in the growth stage, the model achieved *P*, *R*, *AP*, and *FPS* scores of 0.950, 0.967, 0.972, and 87, respectively. These results demonstrate that the TrackSolanum model maintains consistent detection performance across both the seedling and growth stages, exhibiting high precision and recall in each instance. This consistency suggests that the TrackSolanum model can effectively recognize SrD plants throughout their various growth stages.

### 4.3 Effect of image quality on detection results

**Figure 9:**
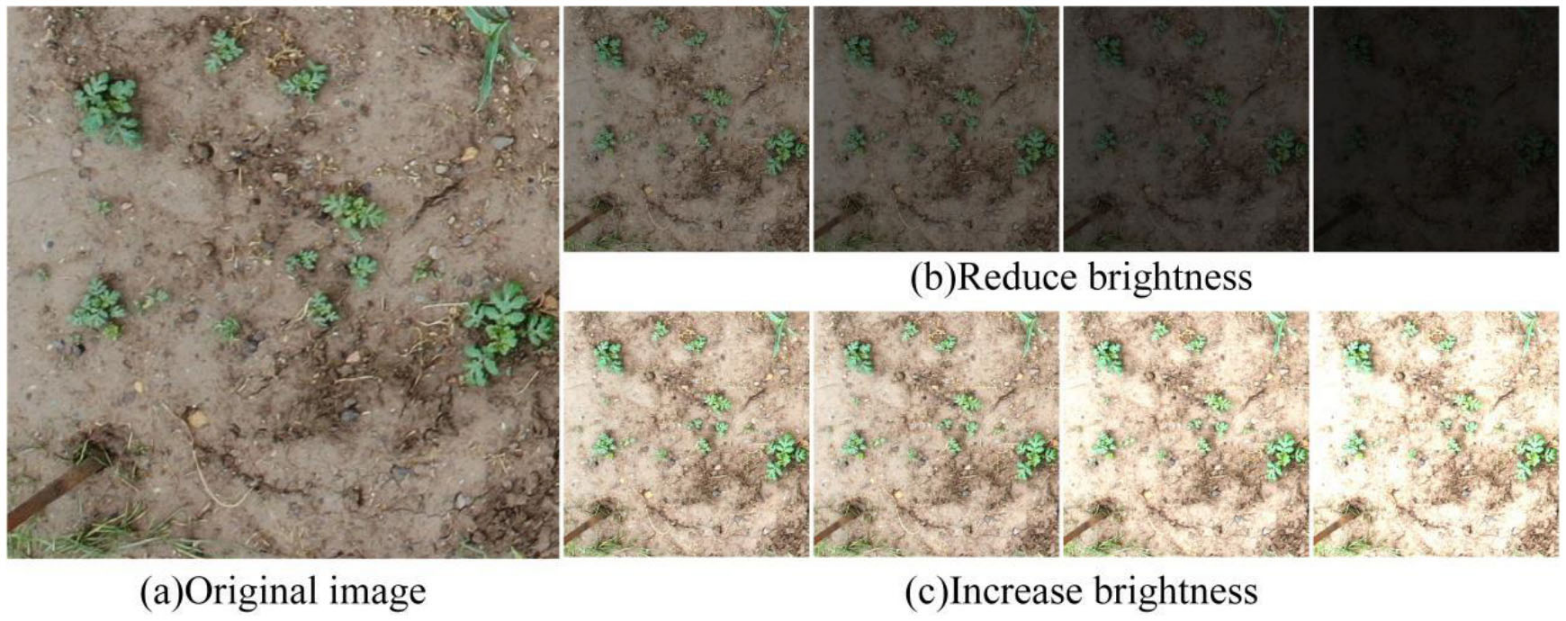
Example of UAV images with varying brightness levels. **(A)** Original image. **(B)** Reduce brightness. **(C)** Increase brightness.

Detection during the early stages of SrD growth holds significant practical importance, as mature plants can establish population dominance, leading to considerable ecological impacts. SrD’s strong environmental adaptability allow it go germinate earlier and grow faster than other plants. At this stage, the contrast with the background is pronounced, and the influence of other vegetation is relatively minimal, creating favorable conditions for detection. Test results show that the model developed in this study performed well in recognizing SrD during both seedling and growing stages. However, real field environments introduce variable such as lighting, which can affect detection accuracy. To assess the model’s performance under low- light conditions, we modified the dataset images to simulate varying lighting conditions by adjusting brightness. The 602 images captured by the UAV at altitudes of 2, 3 and 5 meters were processed. Brightness adjustments were made using a scale factor ranging from 0.2 to 1.8, where a factor of 1.0 indicated no change, factors below 1.0 represented decreased brightness, and factors above 1.0 indicated increased brightness. A total of eight gradients were set (**Figure 9**).

**Table 8** presents the impact of image brightness variation on the model’s detection performance. When the brightness coefficient ranges from 0.8 to 1.0, the model achieves optimal performance across all detection accuracy, with a *P* value of 0.951, *R* value of 0.924, *AP* of 0.984, and an *FPS* of 83. As the brightness coefficient increases or decreases from this range, the model’s performance gradually declines. Under reduced brightness, all evaluation metrics drop. At a brightness coefficient of 0.2, the *P*, *R*, *AP*, and *FPS* fall to 0.913, 0.840, 0.935, and 76, respectively, indicating a higher likelihood of missed detections under low-light conditions. Conversely, as the brightness coefficient increases to 1.8, the model’s detection capabilities diminish significantly, with *P* dropping to 0.890, *R* to 0.756, and *AP* to 0.851. This decline is attributed to overexposure, which impairs the model’s ability to accurately identify targets.

**Table 8:** Impact of image brightness on detection accuracy.

Brightness coefficient	P	R	AP	FPS
0.2	0.913	0.840	0.935	76
0.4	0.938	0.916	0.977	76
0.6	0.943	0.926	0.981	77
0.8	0.952	0.920	0.981	83
1.0	0.951	0.924	0.984	83
1.2	0.943	0.913	0.974	82
1.4	0.943	0.913	0.974	78
1.6	0.909	0.869	0.940	81
1.8	0.890	0.756	0.851	81

**Figure 10:**
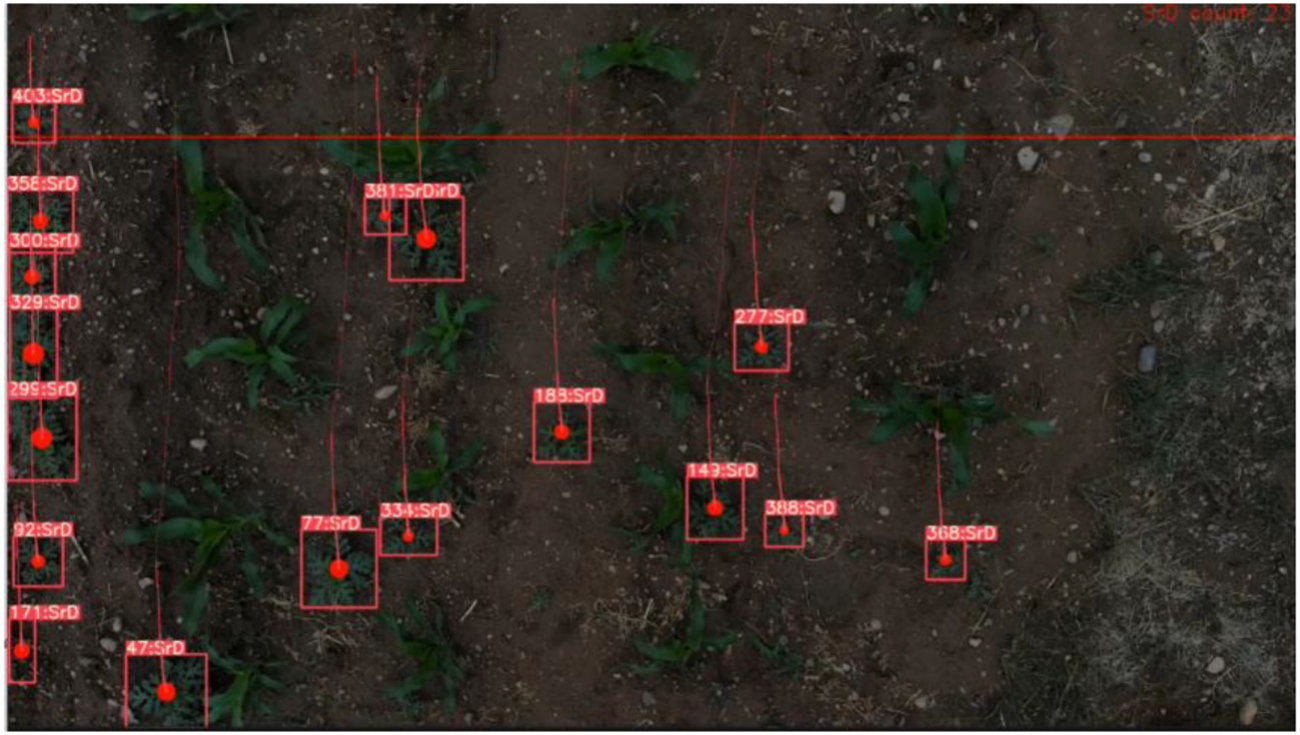
Detection results of the model under low light conditions.

To further assess the impact of lighting conditions on model performance, an additional test was conducted at MP #5 at 6 p.m., when the light levels were lower. The flight height was set to 2 meters. The results of the field test are shown in **Table 9**. As shown in the table, the model still demonstrated a reasonable level of robustness despite the insufficient lighting, with *P*, *R*, *MOTA*, and *IDF1* reaching 0.810, 0.940, 0.617, and 0.870, respectively. However, the lack of sufficient light had a negative impact, leading to a decrease in detection accuracy and errors during tracking, with the *IDSW* reaching 4. The Ground truth of SrD plants in the test area was 58, while the model counted 50 plants, resulting in an ER of 13.793%. **Figure 10** illustrates the results of the field test. The lighting conditions in the field were relatively poor, similar to the simulated test in which the brightness variation coefficient was set to 0.4, though slightly lower. At this point, the model’s precision (P) was 0.810, lower than the 0.938 observed in the simulation. Recall (R) was 0.940, higher than the simulation result of 0.916. Comparing the simulation and field test results, the model’s detection performance for SrD plants remained consistent. This indicates that the model maintained relatively high performance even under low-light conditions.

**Table 9:** Field test results in low light conditions.

Flight Altitude	Time	*P*	*R*	*MOTA*	*IDF1*	*IDSW*	Ground truth	Model count	*ER*/%	*FPS*
2m	16s	0.810	0.940	0.617	0.870	4	58	50	13.793	63

**Figure 11:**
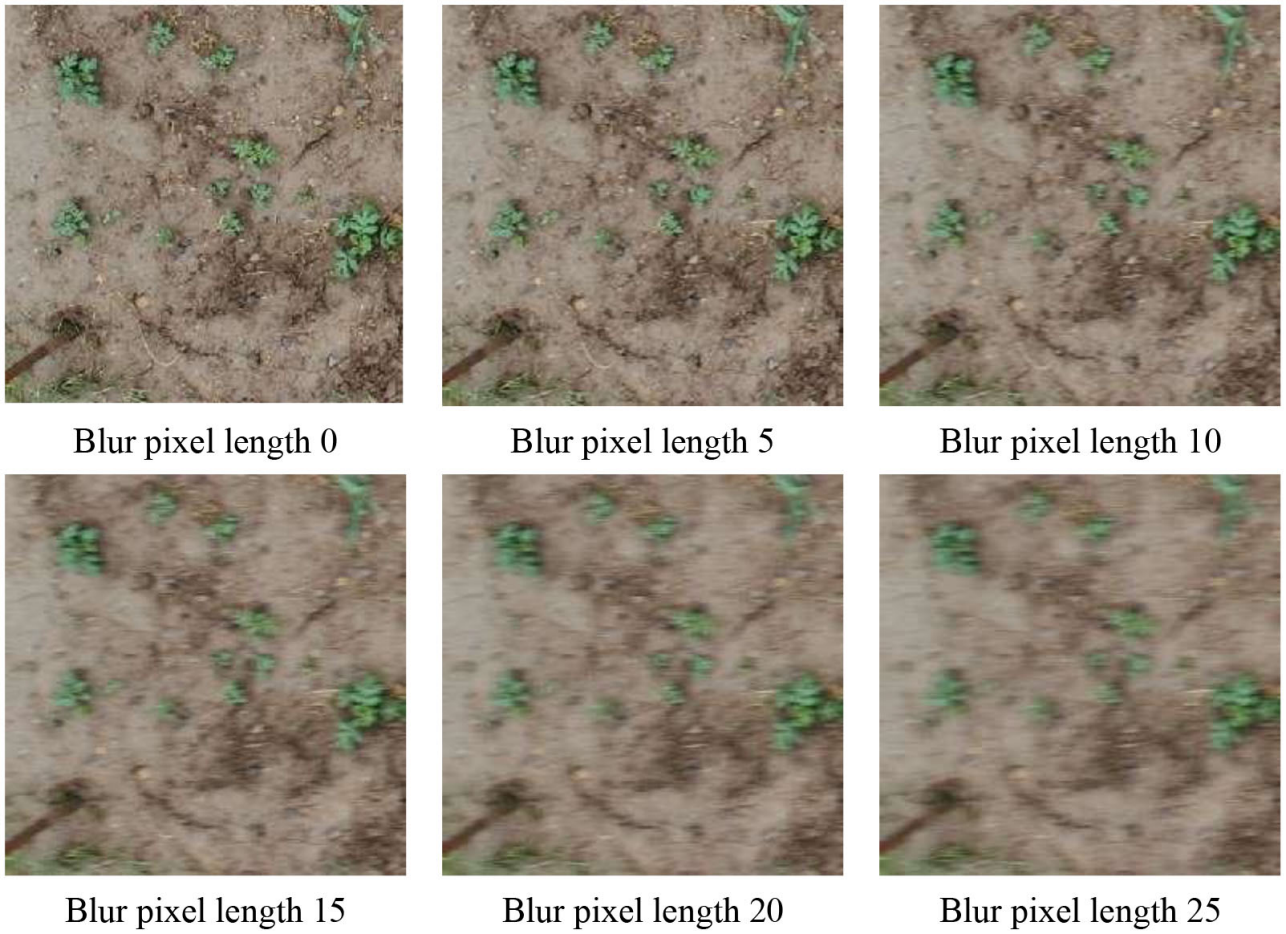
Effect of motion blur pixel length in UAV images.

When UAVs are used to capture videos or images for SrD detection, high flight speeds can result in motion blur in the images. To assess how motion blur affects model performance, 602 images were collected from UAV flights at altitudes of 2, 3, and 5 meters. Six different blur pixel lengths, ranging from 0 to 25 pixels, were applied to simulate varying degrees of motion blur, mirroring the real-world image quality changes caused by UAV flight (**Figure 11**). **Table 10** summarizes the model’s detection performance on blurred images. When the blur pixel length is 0-indicating no blur-the model achieves its best results, with a *P* value of 0.951, *R* value of 0.924, and an *AP* of 0.984, along with an *FPS* of 83, showcasing excellent detection performance. As the blur pixel length increases, the model’s detection accuracy gradually declines. Notably, at blur lengths of 20 and 25 pixels, *P* drops to 0.678 and 0.723, *R* falls to 0.688 and 0.463, and *AP* decreases to 0.749 and 0.628, indicating that motion blur significantly impacts the model’s detection capability. Although *FPS* slightly increases with greater blur lengths-reaching 86 and 91, this comes at the expense of a substantial reduction in detection accuracy and recall. In general, as blur pixel length increases, both *P* and *R* exhibit a declining trend. When the blur length surpasses 20 pixels, the model’s detection quality deteriorates markedly. This underscores that motion blur, especially at higher levels, can significantly impair the model’s ability to accurately localize and identify targets during UAV flights.

**Table 10:** Impact of motion blur on UAV image detection.

Blur pixel length	*P*	*R*	*AP*	*FPS*
0	0.951	0.924	0.984	83
5	0.922	0.918	0.974	85
10	0.879	0.859	0.944	84
15	0.895	0.710	0.866	84
20	0.678	0.688	0.749	86
25	0.723	0.463	0.628	91

To further assess the impact of image quality changes caused by flight speed on model performance, tests were conducted at MP6# with the UAV flying at a height of 3 meters. Video data were captured at three speeds: 0.5 km/h, 1.0 km/h, and 1.5 km/h. The test results are shown in **Table 11**. As the flight speed increases, both *P* and *R* slightly decrease. At 0.5 km/h, the values of *P* and *R* are 0.941 and 0.946, respectively. When the speed increases to 1.0 km/h and 1.5 km/h, the value of *P* drops to 0.935 and 0.916, respectively, while *R* decreases to 0.942 and 0.933. The primary reason for the decline is the loss of image details caused by higher speeds, which significantly impacts small target detection accuracy, especially at higher speeds. Regarding tracking performance, *MOTA* and *IDF1* decrease as the flight speed rises. At 0.5 km/h, the values of *MOTA* and *IDF1* are 0.830 and 0.915, indicating high tracking accuracy. At 1.0 km/h and 1.5 km/h, *MOTA* decreases to 0.817 and 0.776, while *IDF1* drops to 0.910 and 0.901. The increased speed accelerates the relative motion between targets, complicating the frame-to-frame matching process and increasing the miss-match rate. However, overall tracking performance remains high. In the counting task, the increase in flight speed also affects counting accuracy. At 0.5 km/h, 1.0 km/h, and 1.5 km/h, the *ER* is 4.859%, 5.668%, and 9.312%, respectively. Faster speeds lead to more missed or incorrect detection, negatively impacting the final count. Additionally, *IDSW* fluctuates slightly with increasing speed, with values of 32, 33, and 30 at 0.5 km/h, 1.0 km/h, and 1.5 km/h, indicating some instability in tracking performance. Despite these variations, the FPS remains within the range of 79 to 81, demonstrating that the TrackSolanum model can maintain real-time detection and tracking across all tested speeds. This underscores the model’s efficiency and effective use of resources.

**Table 11:** Effect of different flight speeds on model performance.

Flight Speed	Time	*P*	*R*	*MOTA*	*IDF1*	*IDSW*	Ground truth	Model count	*ER*/%	FPS
0.5km/h	60s	0.941	0.946	0.830	0.915	32	247	235	4.859	79
1.0km/h	33s	0.935	0.942	0.817	0.910	33	247	233	5.668	79
1.5km/h	21s	0.916	0.933	0.776	0.901	30	247	224	9.312	81

### 4.4 The significance of enriching the dataset with noisy images

In practical applications, noise is often unavoidable, as it interferes with the effective signals obtained (Mercorelli, 2007(a); Mercorelli, 2007(b) Mercorelli, 2013). Some factors such as lighting, motion, and equipment operation can introduce noise into the captured images during image acquisition. Therefore, this study superimposed three noise signals onto the dataset images to simulate interference caused by different scenarios.In this study, the model was trained using two datasets: one containing noisy images and the other without noisy images. The trained models were then tested on the same test set to verify whether enriching the dataset with noisy images enhances the model’s detection capability. The test results are shown in **Table 12**. After adding noisy images to the training dataset, the model’s P-value increased from 0.932 to 0.947, the R-value improved from 0.931 to 0.944, and the AP-value rose from 0.977 to 0.981. This indicates that accounting for noise during training enables the model to better distinguish between targets and the background, reducing false positives and missed detections, and improving overall performance. Therefore, incorporating noisy images into the training dataset enriches the sample diversity and helps enhance the model’s performance.

**Table 12:** The impact of including noisy images in the training dataset on model performance.

Training set	Number of testing images	*P*	*R*	*AP*	FPS
Without noisy images	1007	0.932	0.931	0.977	98
Include noisy images	1007	0.947	0.944	0.981	98

To further verify the impact of adding noisy images to the dataset on the model’s tracking performance, the model was trained using a dataset without noisy images. It was then tested on UAV videos captured at location MP5 #, with the results shown in **Table 13**. A comparison of **Table 13** and **Table 3** reveals that when the model is trained on a dataset without noisy images, its performance metrics, except for FPS, decrease when detecting the same video. The improvement in FPS can be attributed to the following reason: the model’s detection speed is inversely proportional to the number of SrD plants detected per frame. The more plants detected, the more positional information the model needs to process. When the model’s detection capability declines, the number of detected plants decreases, resulting in less positional information to process, thereby improving detection speed. Training the model on a dataset without noisy images results in decreased detection accuracy, which increases the likelihood of missed detections and false positives, directly affecting the MOTA performance. Furthermore, the decline in detection accuracy can cause temporary target loss or confusion between multiple targets, compromising tracking continuity and accuracy, and leading to an increase in IDSW. These comparison results indicate that incorporating noisy images into the training dataset has a positive impact on improving the model’s performance.

**Table 13:** Results of detecting the video captured at MP5# using a model trained on a dataset without noisy images.

Video	Flight altitude	Time	*P*	*R*	*MOTA*	*IDF1*	*IDSW*	*FPS*
1	2m	26s	0.922	0.930	0.731	0.926	32	27
2	2m	25s	0.927	0.966	0.843	0.947	30	23
3	2m	21s	0.968	0.975	0.819	0.968	35	20
4	2m	34s	0.939	0.973	0.845	0.960	47	17
	average	26.5s	0.939	0.961	0.810	0.950	36	22
5	3m	21s	0.876	0.914	0.703	0.895	14	47
6	3m	50s	0.793	0.949	0.628	0.850	17	31
7	3m	14s	0.845	0.901	0.751	0.878	6	64
8	3m	10s	0.813	0.927	0.690	0.880	5	64
	average	23.75s	0.832	0.923	0.693	0.876	11	52

### 4.5 Analysis of field test results

**Figure 12:**
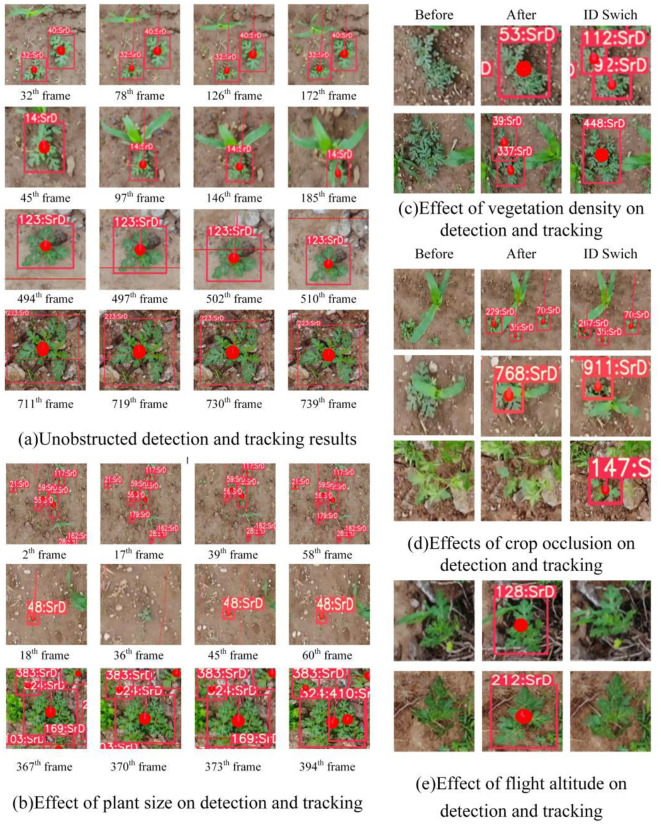
On-site test results for SrD in different situation. **(A)** Unobstructed detection and tracking results. **(B)** Effect of plant size on detection and tracking. **(C)** Effect of vegetation density on detection and tracking. **(D)** Effect of crop occlusion on detection and tracking. **(E)** Effect of flight altitude on detection and tracking.

During field tests, it was noted that vegetation density, plant size, the degree of occlusion, and flight altitude had a significant impact on detection, tracking, and counting results (**Figure 12**). Detection and tracking of SrD are more accurate when it is isolated from other plants (**Figure 12A**). For small SrD seedling, tracking failures or missed detections can occur in certain video frames, Conversely, when the plant is large, a single SrD may be mistakenly identified as two separate plants (**Figure12B**). In cases of dense SrD growth, multiple plants might be detected as a single one, potentially causing ID switches (**Figure12C**). Tracking errors or ID switches are also common when other crops obtruct the SrD, as illustrated in **Figure 12D**). Additionally, at lower drone flight altitudes, rotor interference can affect SrD plants with large leaves, causing them to sway. Excessive leaf movement may lead to failures or undetected targets (**Figure 12E**).

### 4.6 Applicability analysis of the trackSolanum network model

The TrackSolanum network was used to detect, locate, and count SrD in UAV-captured videos taken at various altitudes. The results highlight the TrackSolanum network’s real-time performance and efficiency. In this study, we selected the RGB color model for weed identification. RGB is the default format for UAV-acquired images, simplifying data processing by eliminating the need for color space conversion. Moreover, most computer vision algorithms and deep learning models are commonly trained on RGB images, making them well-suited for the image processing methods applied in this research. Although other color models, such as HSV or HSI, offer benefits in handling hue and luminance variations, our experiments showed that RGB images provided sufficient color and luminance information in real-world environments. Additionally, the high resolution of RGB images ensures better compatibility and flexibility, combined with low data acquisition and processing costs. Implementing the TrackSolanum model offers notable advantages in resource allocation and cost reduction. Firstly, the model minimizes the need for manual inspection and monitoring, optimizing the use of human resources. Secondly, the precise data generated by the model enables more informed decisions regarding herbicide application and mechanical weed control. From a cost perspective, TrackSolanum lowers labor demands by automating inspection tasks, reducing labor expenses, and alleviating pressure on farmers and businesses.

Several factors must be considered when deploying the TrackSolanum network model in real-world conditions. First, the DeepSort algorithm is sensitive to the UAV’s speed and flight path. Although it can handle some camera shake and partial occlusion, high flight speeds or unstable motion may result in tracking loss or ID switching. Second, in large-scale agricultural settings, TrackSolanum’s performance can be constrained by weather, flight altitude, and UAV battery life; poor weather conditions may affect system stability, while higher altitudes can reduce detection accuracy. Third, in densely vegetated areas or when other crops cause occlusions, TrackSolanum may experience false detections and ID switching. Fourth, RGB images are highly sensitive to lighting conditions. Bright light, shadows, and varying angles of illumination can impact image quality, affecting detection accuracy. In complex environments or areas with dense vegetation, targets with similar colors may be challenging to differentiate.

The future work plan includes the following: First, to support real-time processing, efforts will focus on optimizing the computational efficiency of the TrackSolanum model, enabling it to handle longer video streams or higher frame rates without encountering performance bottlenecks. The model will be deployed on UAV or unmanned vehicles through optimized algorithm structures and computational resource allocation. The next step is to incorporate multispectral data to identify targets that are difficult to detect in RGB images, thereby enhancing the model’s robustness and accuracy. Third, continue to explore how to further improve the generalization ability of the model, especially in terms of acquiring more diverse datasets, in order to better adapt it to new detection tasks. Fourth, the model will be tested and optimized in different crops and different environments to improve its adaptability in various complex scenarios. Fifth, propose more advanced localization techniques to solve the problem of locating the unique coordinates of the SrD in the physical world. Through these efforts, the TrackSolanum model is expected to achieve wider application and higher effectiveness in the future.

## 5 Conclusion

This paper proposes a deep learning model, TrackSolanum, capable of real-time detection, localization, and counting of the invasive weed SrD. The TrackSolanum model comprises a detection module YOLO_EAND, a tracking module DeepSort, a localization module, and a counting module. It can detect SrD plants in video streams, assign unique identity IDs, and output their coordinate locations in each image frame image. The YOLO_EAND detection module was tested on SrD at various growth stages. The experimental results indicated that at the seedling stage, the *P*, *R*, *AP* and *FPS* of the YOLO_EAND module reached 0.954, 0.986, 0.990 and 120, respectively. During the growth stage, these metrics were 0.950, 0.967, 0.972 and 87. The results demonstrate that YOLO_EAND module is effective for accurate detection of SrD at different growth stages.

Field validation was conducted using drone-captured videos. The results showed that at a flight height of 2 meters, the SrD detection performance metrics, *P* and *R*, reached a maximum of 0.955 and 0.985, respectively. At this altitude, the tracking performance metrics, *IDSW*, *MOTA*, and *IDF1* were 43, 0.861 and 0.972, respectively, with a counting error rate of only 0.849% and an *FPS* of 13. For the video captured at 3 meters, the *P* and *R* of the TrackSolanum model reached 0.941 and 0.946, while the tracking performance metrics *IDSW*, *MOTA*, and *IDF1*, were 32, 0.830 and 0.915, respectively, with a counting error rate of 4.859% and an *FPS* of 79. The TrackSolanum network presented in this paper is not only proficient in the on-site detection of the invasive weed SrD but is also well-suited for the real-time detection and processing of various other weeds and crop seedlings.

## Data Availability

The original contributions presented in the study are included in the article/supplementary material. Further inquiries can be directed to the corresponding authors.
